# Chromic soft crystals based on luminescent platinum(II) complexes

**DOI:** 10.1107/S2052252524003658

**Published:** 2024-06-11

**Authors:** Masako Kato

**Affiliations:** a Department of Applied Chemistry for Environment Kwansei Gakuin University 1 Gakuen Uegahara Sanda Hyogo669-1330 Japan; Université de Sherbrooke, Canada

**Keywords:** soft crystals, luminescence, chromic phenomena, Pt(II) complexes

## Abstract

Platinum(II) complexes of square-planar geometry are interesting from a crystal engineering viewpoint because they exhibit strong luminescence based on the self-assembly of molecular units. The luminescence color changes in response to gentle stimuli, such as vapor exposure or weak mechanical forces. Both the molecular and the crystal designs for soft crystals are critical to effectively generate the chromic luminescence phenomenon of Pt(II) complexes. In this topical review, strategies for fabricating chromic luminescent Pt(II) complexes are described from a crystal design perspective, focusing on the structural regulation of Pt(II) complexes that exhibit assembly-induced luminescence via metal–metal interactions and structural control of anionic Pt(II) complexes using cations. The research progress on the evolution of various chromic luminescence properties of Pt(II) complexes, including the studies conducted by our group, are presented here along with the latest research outcomes, and an overview of the frontiers and future potential of this research field is provided.

## Abstract 2

Platinum(II) complexes exhibit intense luminescence based on their molecular arrangement and chromic luminescence, which is a color change in response to gentle stimuli such as vapor exposure or weak mechanical forces. Both the molecular and the crystal designs for soft crystals are critical to effectively control the chromic phenomenon of platinum(II) complexes.

## 1. Introduction

The development of crystalline materials that exhibit interesting photofunctions has made remarkable progress beyond the frameworks of organic, inorganic and metal-complex crystals. In particular, flexible response systems have attracted considerable attention in the form of soft crystals (Kato *et al.*, 2019 ▸; Kato & Ishii, 2023 ▸). Generally, the term ‘soft’ in solid materials is used for materials that can be easily deformed near room temperature. Polymers, gels and some metals may be included in such soft materials. The soft crystals we focus on here refer to highly ordered crystalline materials that respond to gentle stimuli while maintaining their three-dimensional order as crystals and changing their visible properties. Some soft crystals respond to chemical vapor, whereas others react to mechanical stimuli such as rubbing and scratching, leading to remarkable changes in color and luminescence. These visible changes are based on structural transformations and phase transitions, occurring reversibly in response to gentle stimuli. The reversible phenomena of color change are termed chromism and include thermochromism, photochromism, mechanochromism and vapochromism. These materials are expected to show promising applications as highly sensitive environmental sensors and probes. Additionally, structurally flexible and highly ordered systems are excellent targets for developing an in-depth understanding of the chromic phenomena, dynamics of phase transformations and excited-state structural changes.

This study focuses on luminescent Pt(II) complexes exhibiting chromic phenomena (see Scheme[Chem scheme1] below), thus acting as soft crystals. Pt(II) complexes with a square-planar coordination geometry can offer various strategies for unique molecular and crystal structural designs to construct chromic systems with high emission efficiency and sensitivity. Simple planar structures enable different molecular arrangements, exhibiting characteristic properties of the assembled systems which can be finely tuned using molecular design. Herein, the latest studies, including their background, are reviewed in two parts. In Section 2, Pt(II) complexes exhibiting assembly-induced luminescence are discussed, along with the color control by stacking structures, vapochromic single-crystal-to-single-crystal (SCSC) transformations and chromic triboluminescence. Section 3 describes the luminescent Pt(II) complexes controlled by counterions. Some anionic Pt(II) complexes combined with counter cations show highly efficient luminescence, whereas others exhibit unique ionic liquid properties.
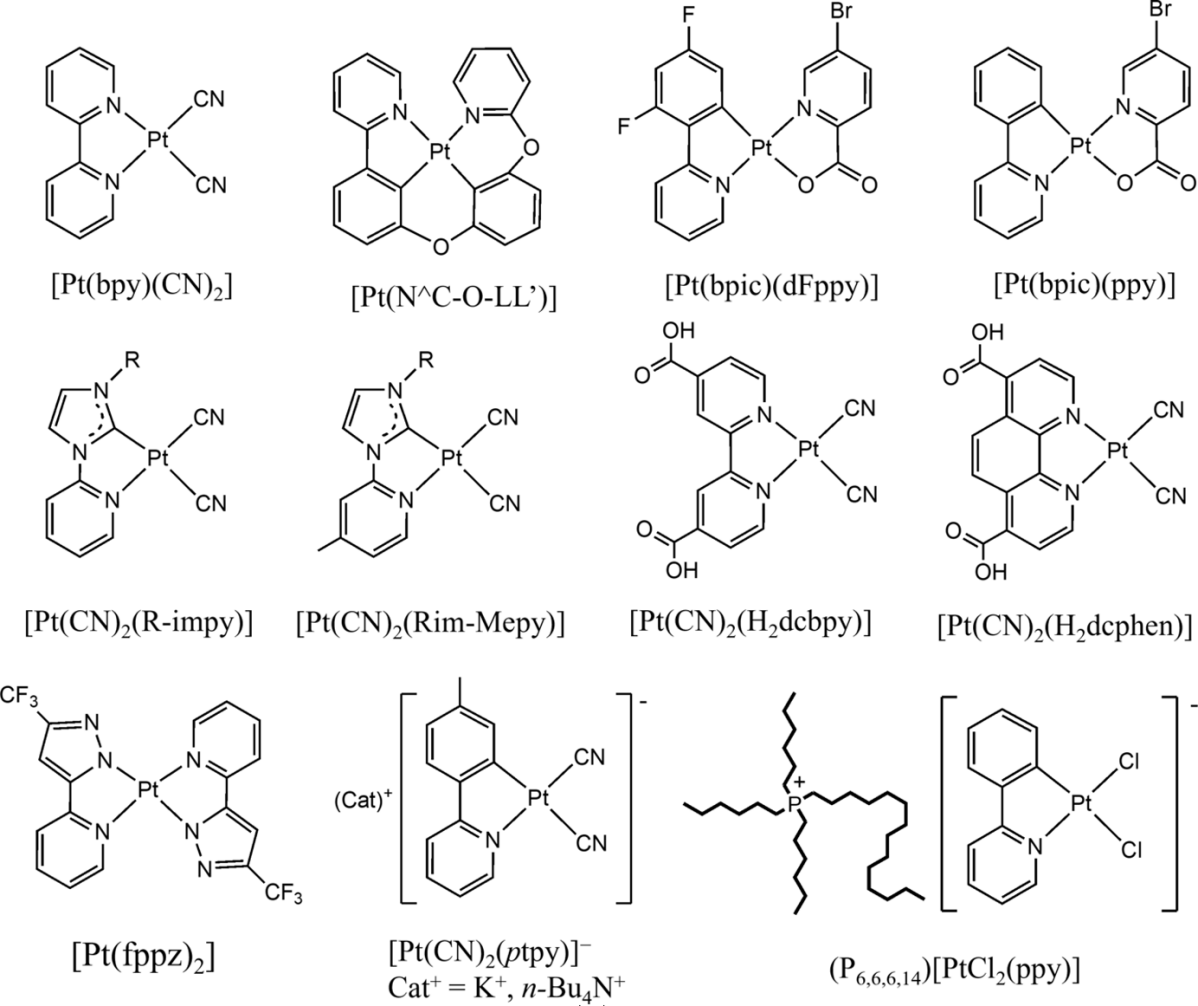


## 2. Pt(II) complexes exhibiting assembly-induced luminescence

### 2.1. Color control of stacking structures

Divalent platinum complexes typically adopt a square-planar coordination geometry. The planar complex units show stacking owing to Pt–Pt and/or ligand π–π interactions, which exhibit characteristic luminescence (Yam *et al.*, 2015 ▸; Aliprandi *et al.*, 2016 ▸). This self-assembly competes with the intermolecular interactions between the complexes and solvent molecules in solution, which are dependrnt on the concentration and the nature of the solvent. Further, the physical properties of self-assembled states are ambiguous owing to the large fluctuations, but as the self-assembly progresses due to complicated intermolecular interactions, large aggregates are formed to generate the solid state. In the crystalline state, the atomic arrangement can be precisely controlled, facilitating the development of characteristic optical functions. In metal complexes, various intermolecular interactions such as hydrogen bonds, inter-ligand π–π and CH–π, halogen–halogen, and metal–metal interactions can be utilized to control the assembled structures (Gu *et al.*, 2023 ▸; Dutta *et al.*, 2022 ▸). Recently, Zhong and coworkers reported a series of Pt(II) complexes with cruciform shapes that exhibited phospho­rescence of different colors controlled by two-dimensional packing structures in crystals based on various intermolecular π−π interactions (Xu *et al.*, 2022 ▸). Thus, the intermolecular interactions expand the structural diversity, but these systems remain challenging in terms of the emission quantum yield (Φ). In contrast, the assembled structures with metal–metal interactions have the advantage of constructing systems with high emission efficiency and luminescence color tunability.

**Figure 1:**
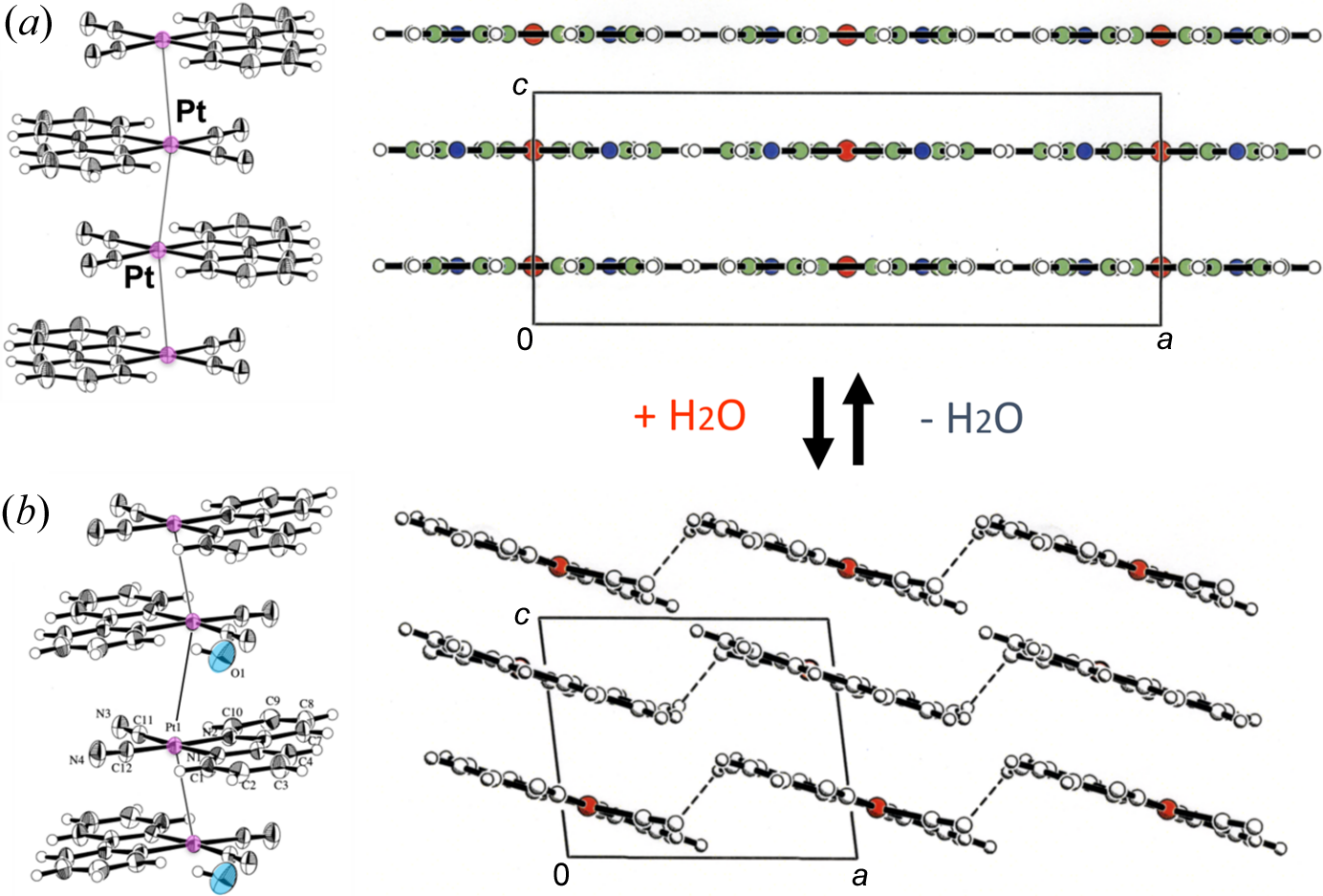
Stacking and packing structures of [Pt(bpy)(CN)_2_]: (*a*) red anhydrous and (*b*) yellow monohydrate forms. Reprinted (adapted) with permission from Kato (2007 ▸). Copyright (2007) Chemical Society of Japan.

**Figure 2:**
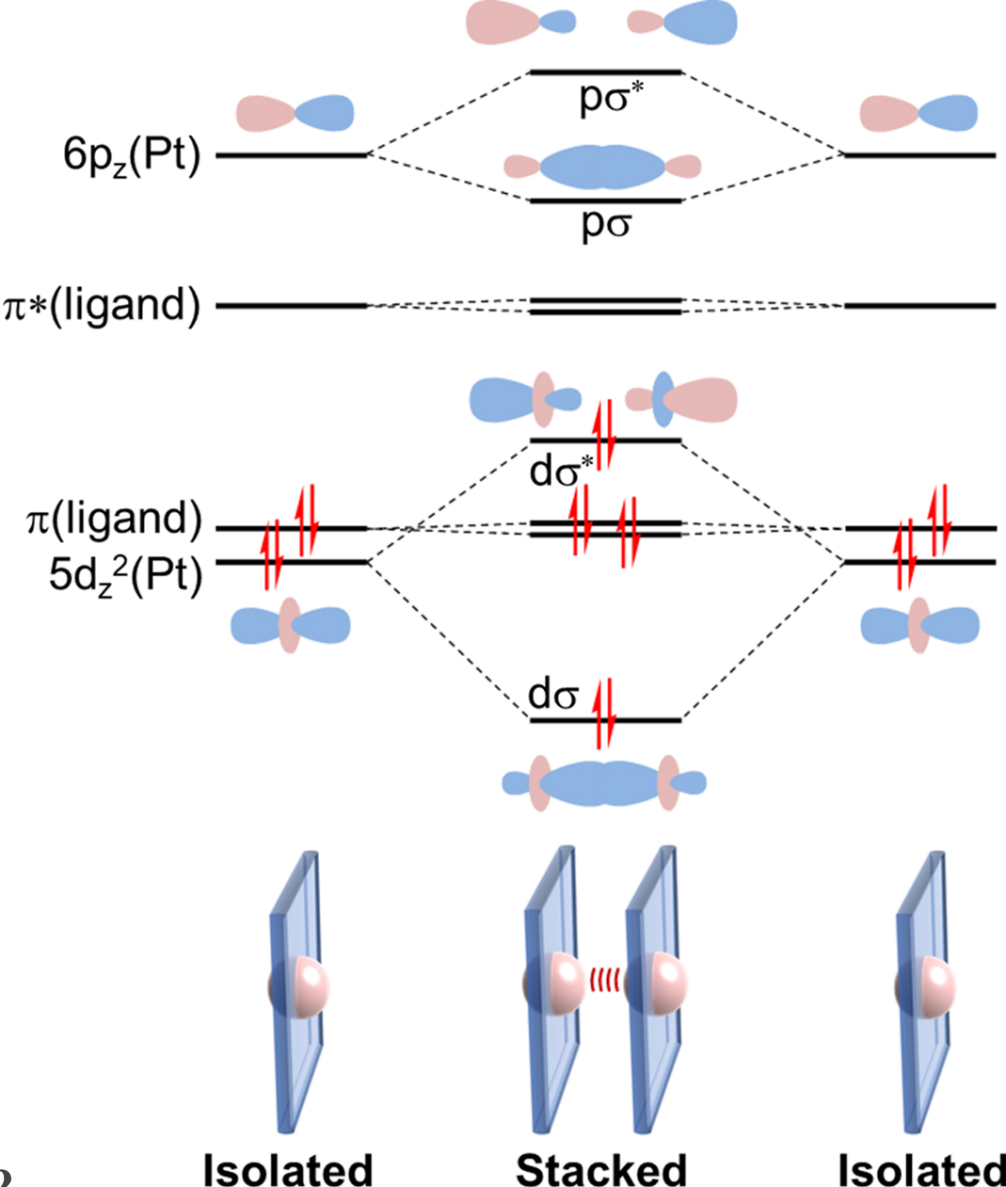
Schematic molecular orbital energy diagram for the isolated and stacked Pt(II) complexes. Reprinted from Yoshida & Kato (2018 ▸), Copyright (2018), with permission from Elsevier.

**Figure 3:**
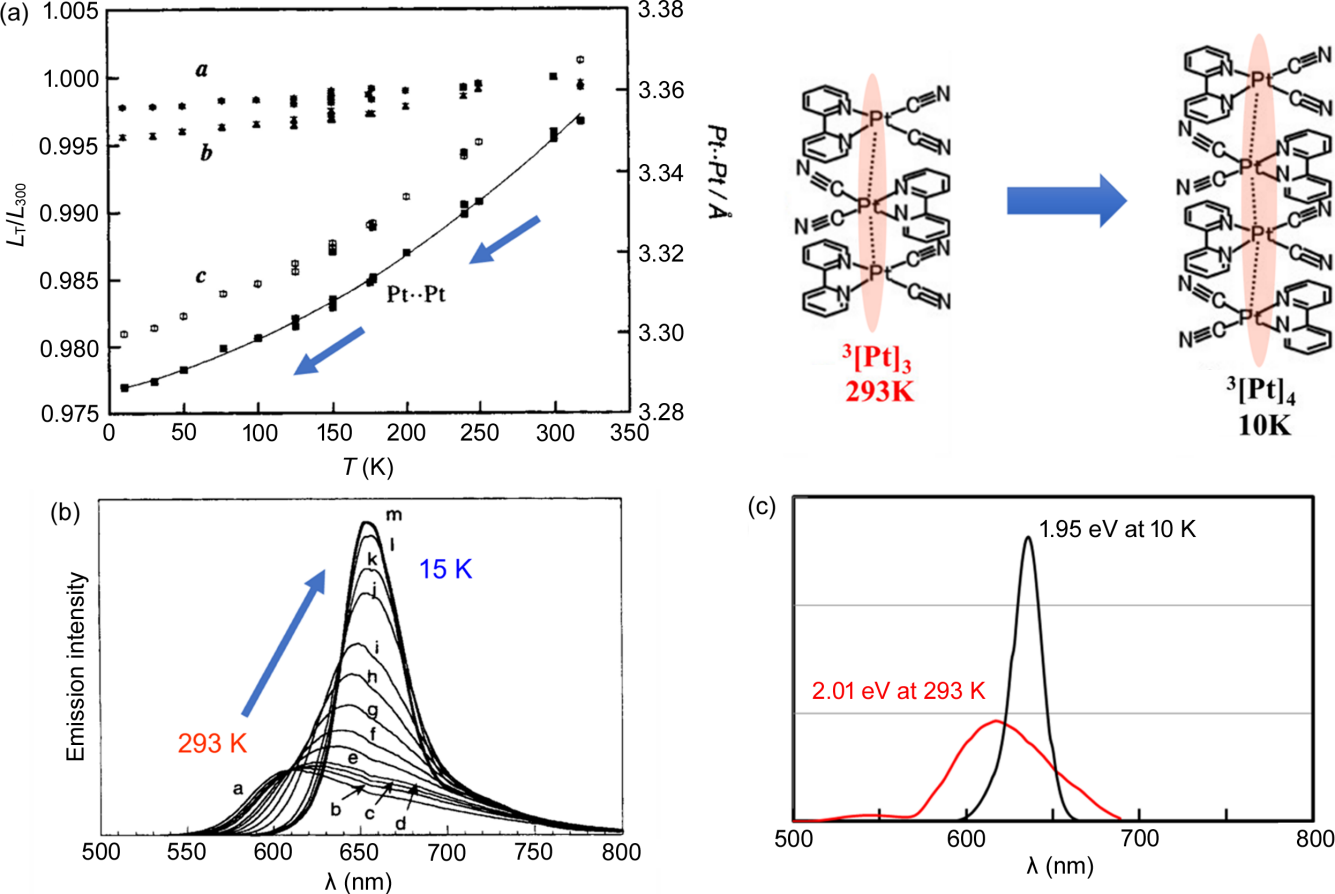
Red form of [Pt(bpy)(CN)_2_]. (*a*) Ratio of cell parameters (*a*, *b* and *c*) relative to those at room temperature (300 K) and the Pt⋯Pt distance plotted as a function of temperature. (*b*) Emission spectra at different temperatures: a = 292 K, b = 260 K, c = 240 K, d = 220 K, e = 180 K, f = 160 K, g = 140 K, h = 120 K, i = 100 K, j = 60 K, k = 45 K, l = 30 K, m = 15 K; λ_ex_ = 514.5 nm. (*c*) Calculated ^3^MMLCT emission spectra for the trimer and tetramer of [Pt(bpy)(CN)_2_]. Parts (*a*) and (*b*) reprinted (adapted) with permission from Kato *et al.* (1999 ▸). Copyright (1999) American Chemical Society. Part (*c*) reprinted (adapted) with permission from Nakagaki *et al.* (2020 ▸). Copyright (2020) American Chemical Society.

For instance, [Pt(bpy)(CN)_2_] (bpy = 2,2′-bi­pyridine) crystallizes as bright red crystals with intense red luminescence from a colorless solution of *N*,*N*-di­methyl­formamide. For the red form, the complex units are stacked with short Pt⋯Pt contacts, forming a one-dimensional chain [Fig. 1 ▸(*a*)]. The Pt⋯Pt distance [3.348 (2) Å] is sufficiently short to generate Pt⋯Pt electronic interactions (Connick *et al.*, 1996*a* ▸). Conventionally, coloration is rationalized by the overlap of the *d*_*z*^2^_ orbitals in the stacked structure, resulting in an increase of the highest occupied molecular orbital (HOMO) as the *d*σ* orbital (Fig. 2 ▸). The transition from the *d*σ* to the π* orbital of the ligand occurs in the visible region as a metal-to-metal-to-ligand charge transfer (MMLCT) transition, and efficient luminescence occurs from the corresponding triplet state as ^3^MMLCT emission in the stacked form. The diagram reveals that the MMLCT transition energy decreases with increasing Pt⋯Pt interaction. The temperature-dependent emission spectral shifts show a good correlation with the Pt⋯Pt distances for the red form of [Pt(bpy)(CN)_2_] and other relevant Pt(II) complexes (Kato *et al.*, 1999 ▸; Connick *et al.*, 1996*b* ▸). Recently, Sakaki and coworkers rationalized the emission energy shift using theoretical calculations of the emission excited states (Nakagaki *et al.*, 2020 ▸). They successfully reproduced the temperature dependence of the emission spectra based on their periodic QM/MM study, wherein the emission spectrum at 293 K could be attributed to the ^3^MMLCT emission from the trimer, and the emission at 10 K to the tetramer (Fig. 3 ▸). The emission spectral red-shift is attributed to the extension of the ^3^MMLCT excited state within the Pt⋯Pt chain. Thus, it is important to note that the Pt⋯Pt distance in the ground state correlates with the expansion of the excited states, which was precisely evaluated for the first time in this one-dimensional stacking system.

For the self-assembled systems, the Pt⋯Pt interactions can be controlled via gentle stimuli such as vapor exposure and weak mechanical forces. For example, the red form of [Pt(bpy)(CN)_2_] exhibits a remarkable color change to yellow on exposure to water vapor, forming a monohydrate. The crystal structure indicates that the yellow form has an oblique stack with inequivalent Pt⋯Pt distances [3.3279 (3) and 4.6814 (3) Å] (Kishi & Kato, 2002 ▸) [Fig. 1 ▸(*b*)], suggesting the Pt⋯Pt interaction weakens in the yellow form compared with the red one. The color reverts from yellow to red on the release of water molecules. This vapor-induced reversible color change phenomenon is called vapochromism (Kato, 2007 ▸; Wenger, 2013 ▸). It has attracted considerable attention for easy sensing of harmful and invisible gaseous substances in the environment. Additionally, vapochromism is of scientific interest owing to the visualization of interactions between solids and gasses. Several vapochromic materials have been developed and those exhibiting luminescent color changes are visually more attractive and appropriate for sensors (Kato *et al.*, 2022 ▸; Yam & Cheng, 2022 ▸). Complicated changes often occur in chromic complexes that respond to weak stimuli. Recently, in-depth investigations of multi-stimuli responses and multi-chromic phenomena were reported (Ni *et al.*, 2023 ▸; Martínez-Junquera *et al.*, 2022 ▸; Shigeta *et al.*, 2023 ▸; Morimoto *et al.*, 2023 ▸).

Regarding mechanical effects, the elastic properties can be induced along with luminescence properties in Pt(II) complex crystals with anisotropically stacked structures controlled via Pt⋯Pt and additional intermolecular interactions. Co-crystals of [Pt(bpic)(dFppy)] [bpic = 5-bromo­picolinate, dFppy = 3,5-difluoro-2-(pyridin-2-yl)phenyl] and [Pt(bpic)(ppy)] [where ppy = 2-(pyridin-2-yl)phenyl] bent easily under weak mechanical stress (elastic modulus: 1.9 and 2.6 GPa for short and mid-axis directions, respectively), and showed intense orange luminescence that originated from the ^3^MMLCT state (*Φ* = 0.94) (Makino *et al.*, 2023 ▸). Compared with the co-crystal, the respective crystals showed emission quantum yields of less than half. Moreover, the [Pt(bpic)(ppy)] crystal was brittle and fractured by similar mechanical forces, and the [Pt(bpic)(dFppy)] crystal was elastic but exhibited monomer-based ^3^ππ* emission. Considering the trade-off between strong stacking for efficient MMLCT emission and elastic deformability (Yoshida *et al.*, 2021 ▸), the co-crystal is notable for its loosely connected zigzag-strand packing structure.

### 2.2. Red–blue luminescence by fine tuning the MMLCT emission state

**Figure 4:**
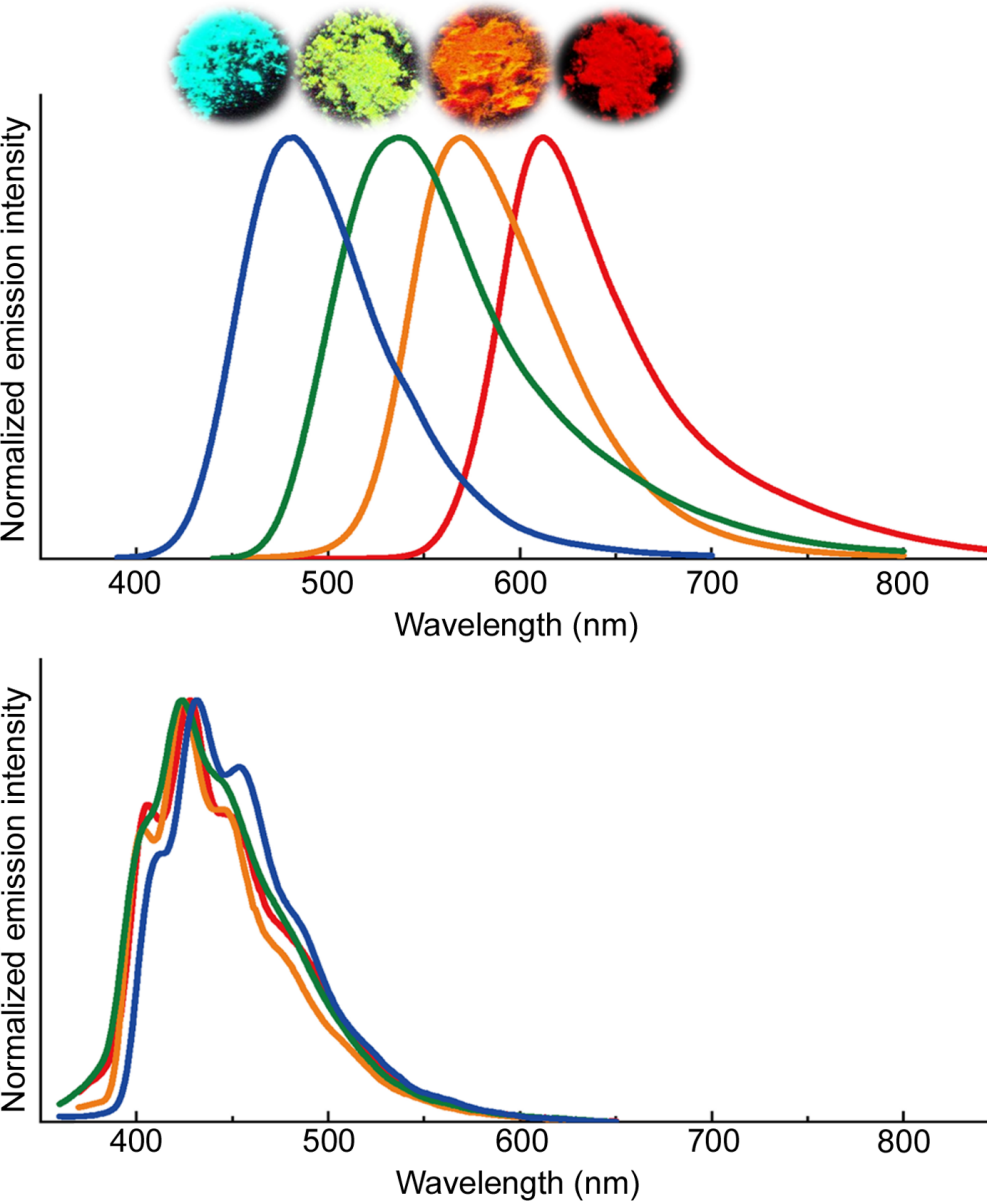
Emission spectra of [Pt(CN)_2_(*R*-impy)] in Pt1-Me (red), Pt1-Et (orange), Pt1-*^i^*Pr (green) and Pt1-*^t^*Bu (blue). (*a*) Solid state at room temperature (λ_ex_ = 350 nm) and (*b*) EtOH–MeOH (1:1 *v*/*v*, 1.0 × 10^−7^ *M*) at 77 K (λ_ex_ = 280 nm). Reprinted with permission from Saito *et al.* (2020 ▸). Copyright (2020) John Wiley & Sons.

As discussed in the previous section, the emission color change based on the MMLCT state is highly sensitive to the stacking structure. However, conventional self-assembling systems have a limited color range, limited to the red–yellow region or green emission (Hsu *et al.*, 2016 ▸). Luminescence in the blue region was primarily observed for monomeric Pt(II) complexes as ligand-centered ππ* emissions; however, the emission intensity was relatively weak for complexes with a tendency to self-assemble. It was challenging to expand the color region of the MMLCT emission to obtain blue emission with a high emission quantum yield at room temperature. To address this issue, the construction of precisely adjusted stacking structures with extremely weak Pt⋯Pt interactions was essential. According to the theoretical calculations, the binding energy of the Pt⋯Pt interactions that causes the self-assembly of Pt(II) complexes has been evaluated to be around 4–10 kJ mol^−1^ for several Pt(II) complexes (Novoa *et al.*, 1995 ▸; Stoyanov *et al.*, 2003 ▸; Pérez Paz *et al.*, 2012 ▸). The values are weaker than those common for hydrogen bonds (10–40 kJ mol^−1^). However, much weaker than the normal Pt⋯Pt interactions and stable stacking are required to achieve efficient blue ^3^MMLCT emission. In this context, to improve the color tunability and emission efficiency, a series of Pt(II) complexes, [Pt(CN)_2_(*R*-impy)], using an *N*-heterocyclic carbene ligand, *R*-impyH^+^ = 1-alkyl-3-(pyridin-2-yl)-1*H*-imidazolium [where *R* = Me (Pt1-Me), Et (Pt1-Et), *^i^*Pr (Pt1-*^i^*Pr) and *^t^*Bu (Pt1-*^t^*Bu)] were synthesized (Saito *et al.*, 2020 ▸). The Pt(II) complexes exhibited intense luminescence (*Φ* = 0.5–0.9) over red–blue on changing the substituent (*R*) from Me to *^t^*Bu. Fig. 4 ▸ shows the emission spectra of the complexes in the solid state at room temperature compared with those in dilute solutions at 77 K. Remarkably, blue ^3^MMLCT emission is observed for the first time in Pt1-*^t^*Bu. In contrast, structured emission spectra with relatively long emission lifetimes (12–15 µs) assigned to the ^3^ππ* emissions of the monomer complexes are obtained in a dilute glassy solution at 77 K. Therefore, the alignment of the complexes in the crystal is crucial for color control using the characteristic ^3^MMLCT luminescence.

**Figure 5:**
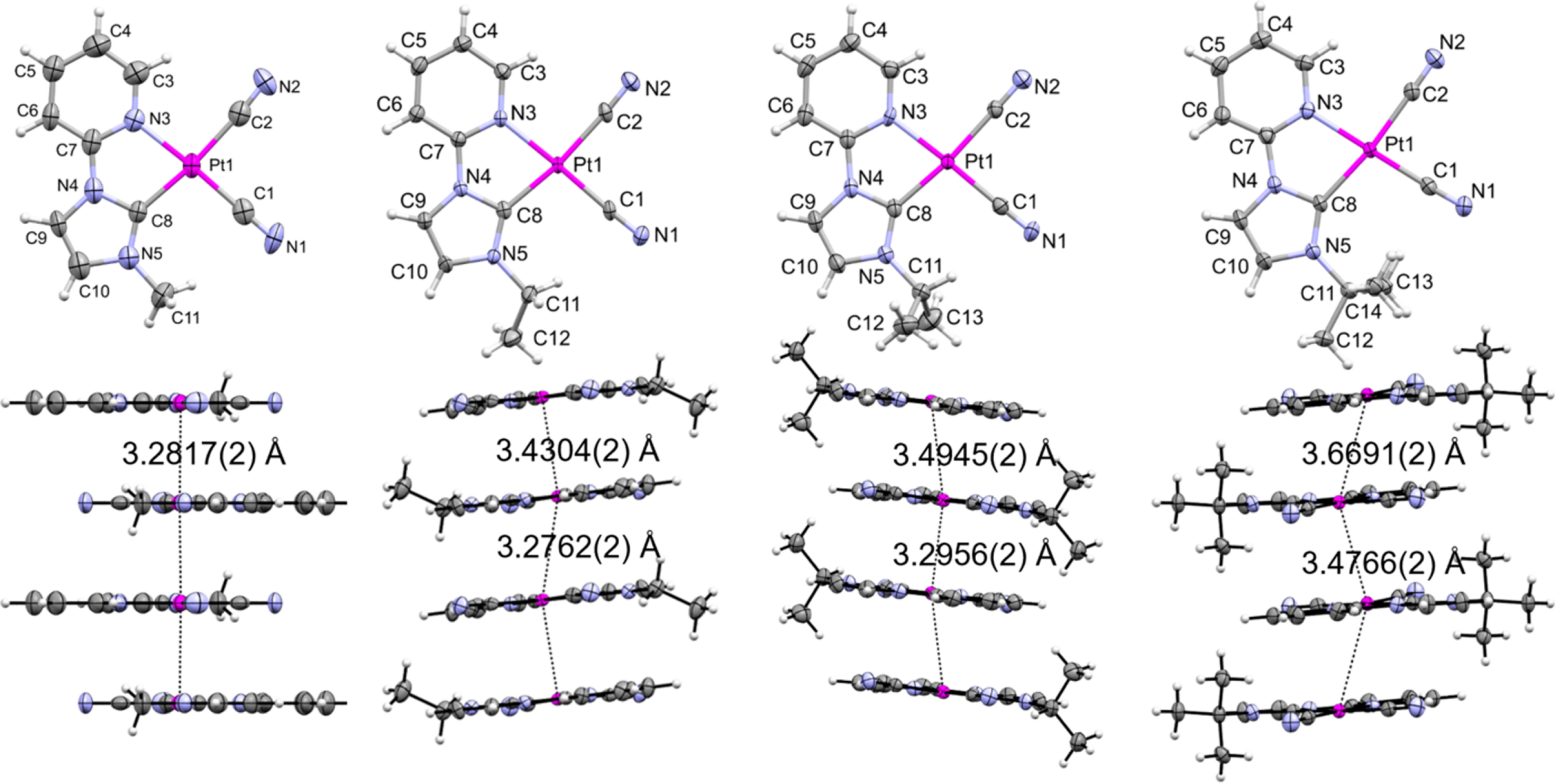
*ORTEP* drawings of the molecular (upper) and stacking structures along the *b* axis (lower) of Pt(II) complexes at 200 K: (*a*) Pt1-Me, (*b*) Pt1-Et, (*c*) Pt1-*^i^*Pr and (*d*) Pt1-*^t^*Bu. Thermal ellipsoids are displayed at a 50% probability level for non-hydrogen atoms. The solvent molecules are omitted for clarity. Reprinted with permission from Saito *et al.* (2020 ▸). Copyright (2020) John Wiley & Sons.

**Figure 6:**
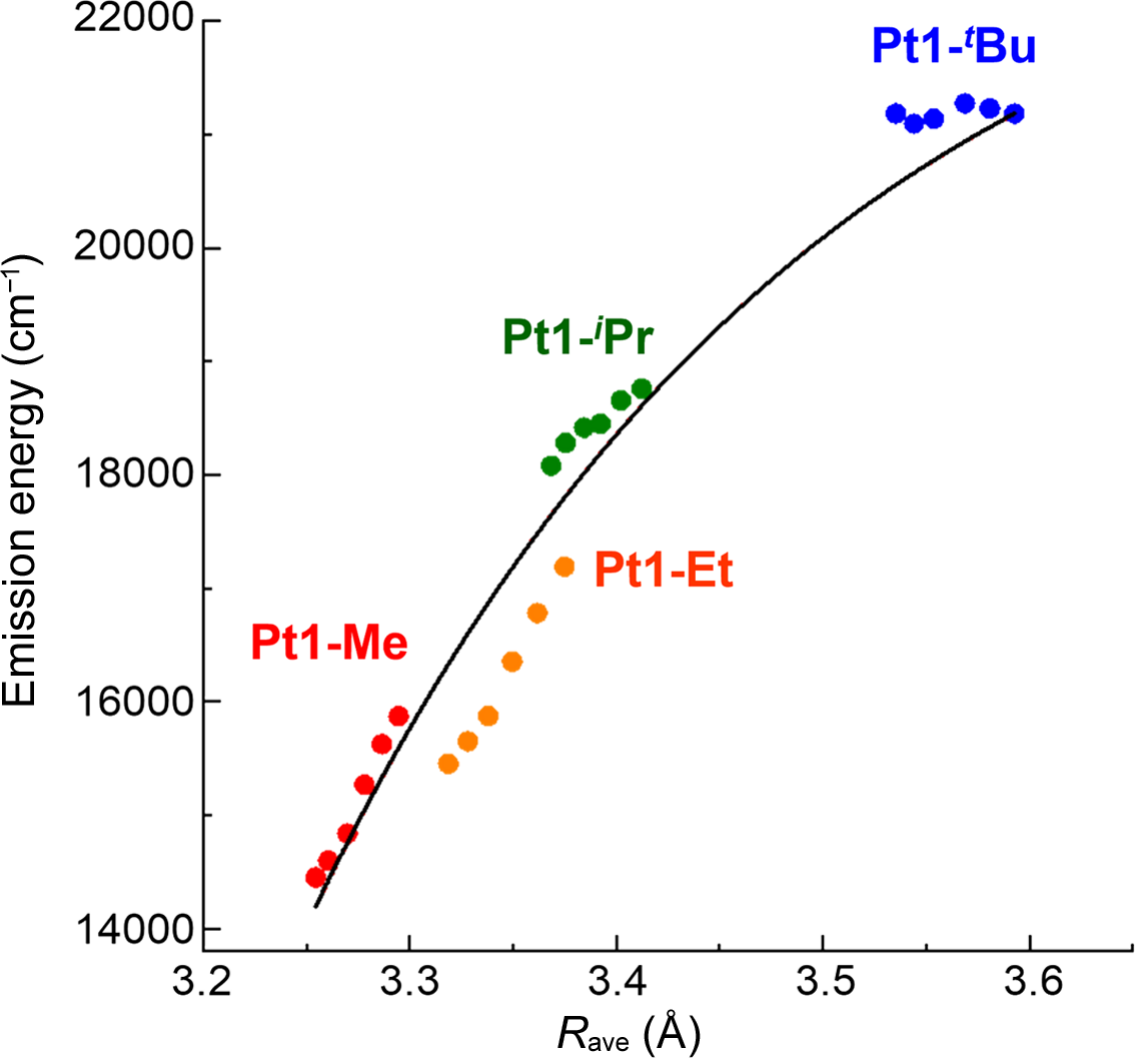
Correlation between maximum emission energies and Pt⋯Pt distances (*R*_ave_) for the four complexes from 100–250 K: Pt1-Me (red), Pt1-Et (orange), Pt1-*^i^*Pr (green) and Pt1-*^t^*Bu (blue). Reprinted with permission from Saito *et al.* (2022 ▸). Copyright (2022) John Wiley & Sons.

Single-crystal X-ray analysis revealed that these complexes form stacking structures in the crystals, but their stacking patterns are slightly different (Fig. 5 ▸). For Pt1-Me, the complexes are stacked parallel, whereas the other complexes exhibit slightly inclined stacks owing to the substituents, and the averaged Pt⋯Pt distance increases from Pt1-Me to Pt1-Et, Pt1-*^i^*Pr and Pt1-*^t^*Bu (in this order), which is consistent with the color change from red to blue. The temperature dependences of the emission spectrum and crystal structure were investigated, which provided essential information regarding Pt⋯Pt interactions. The results are given in Fig. 6 ▸ as emission energy versus averaged Pt⋯Pt distance (*R*_ave_) plots, which show a correlation within the respective complex and over the four complexes as well. The slope based on the temperature change becomes smaller in the order Pt1-Me > Pt1-Et > Pt1-*^i^*Pr, and in the case of Pt1-*^t^*Bu, the Pt⋯Pt distance changes with temperature, but the emission energy does not. The features of the three complexes except for Pt1-*^t^*Bu are similar to that of [Pt(bpy)(CN)_2_], implying that the emission energy changes with decreasing temperature are ascribed to the expansion of the excited state from dimer, trimer to tetramer in accordance with decreasing Pt⋯Pt distance. However, for Pt1-*^t^*Bu with a weak and inequivalent stack, the excited state would be localized in the dimer. The results confirmed that controlling the stacking manner is essential for color control of the assembly-induced luminescence.

### 2.3. Dynamics of vapochromic transformations

**Figure 7:**
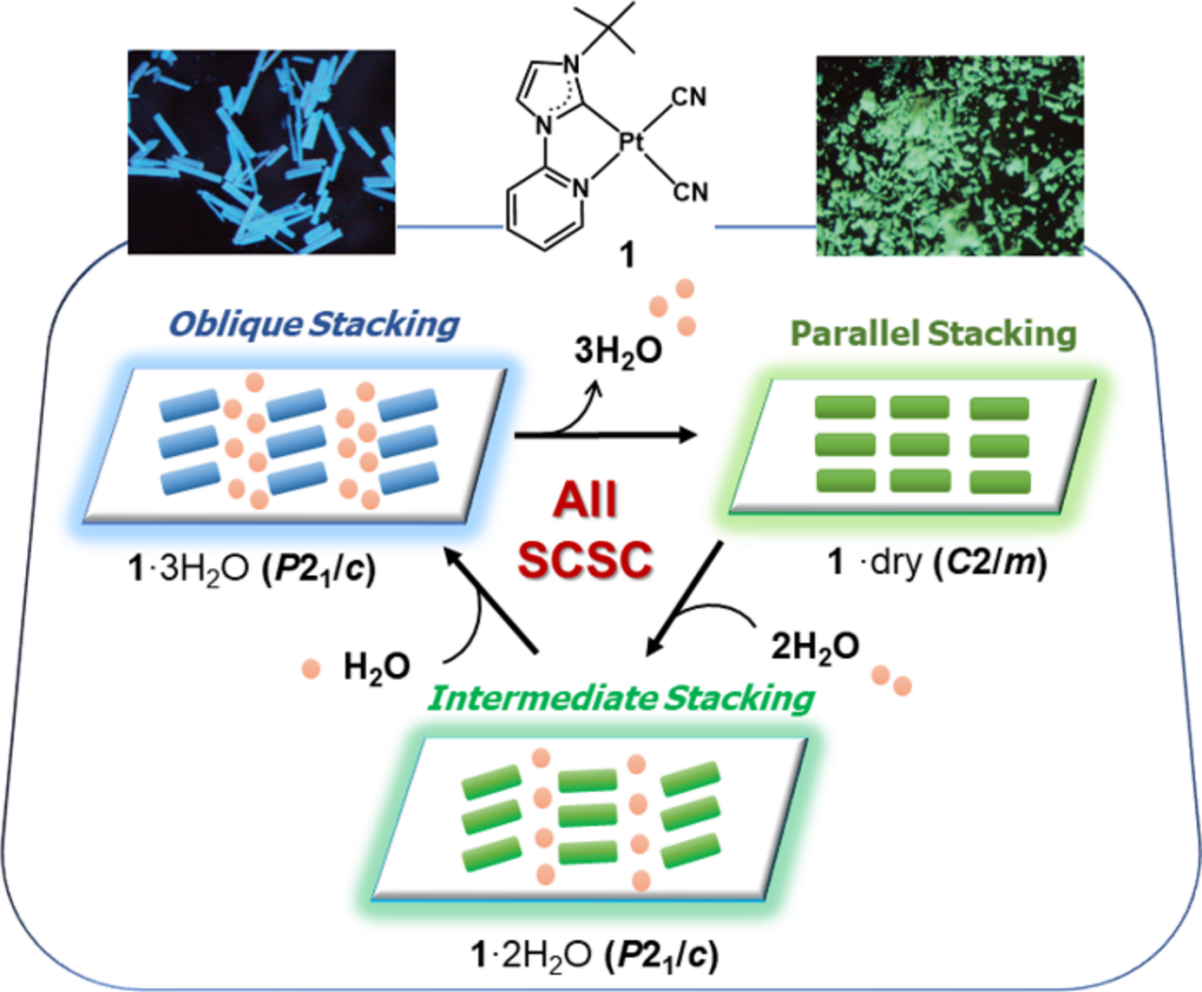
Schematic of the structural changes during the vapor-induced reversible and stepwise SCSC transition of Pt1-*^t^*Bu. Reprinted with permission from Saito *et al.* (2022 ▸). Copyright (2022) John Wiley & Sons.

Weakly stacked systems are flexible and can form good soft crystals. For instance, the previously mentioned blue-luminescent Pt(II) complex, Pt1-*^t^*Bu, exhibits vapochromic behavior in response to water and methanol vapor (Saito *et al.*, 2022 ▸). The blue luminescent form included crystallized water as the trihydrate form, which was stable in sealed vessels. However, after drying *in vacuo*, the luminescence color changed from blue to yellow–green, forming anhydrous crystals, and reverted to trihydrate on water vapor exposure. This phenomenon occurred reversibly while maintaining single crystallinity, which enabled the investigation of dynamic structural changes. *In situ* diffraction measurements under controlled vapor pressure revealed that the SCSC transformation proceeded in two steps from the anhydrous to the trihydrate form via a dihydrate. The first step of dihydrate formation proceeded at a saturated vapor pressure of 3 kPa; however, the second step of trihydrate formation required an air pressure of 100 kPa. The stacking pattern changed stepwise from a parallel to an intermediate stack and subsequently to an oblique stack with the expansion of the intercolumn distances owing to water uptake (Fig. 7 ▸). Thus, it forms an ideal example of vapochromic SCSC transformation.

**Figure 8:**
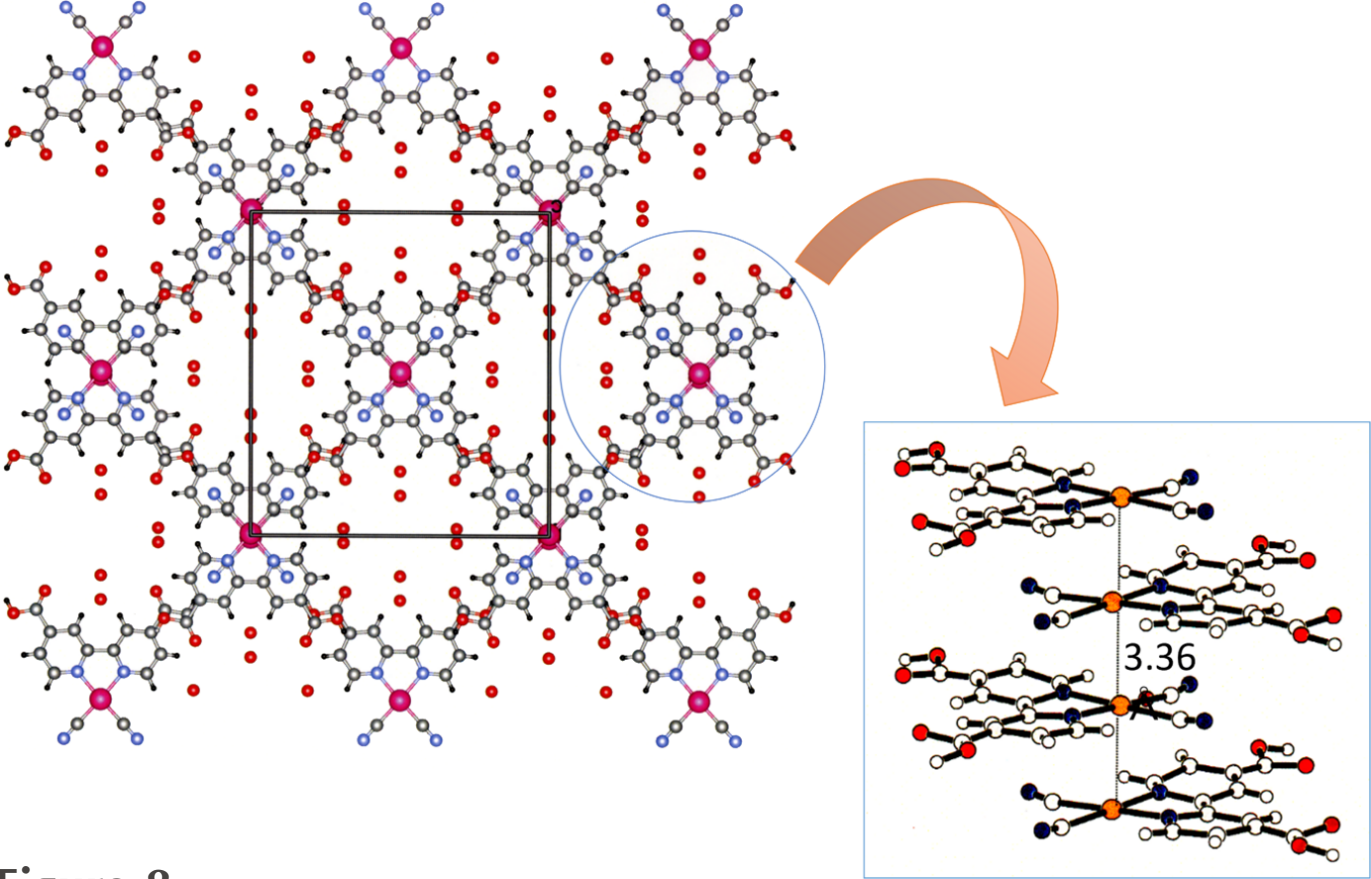
Supramolecular structure of the red [Pt(CN)_2_(H_2_dcbpy)] form along with the stacking structure. Reprinted (adapted) with permission from Kato *et al.* (2005 ▸). Copyright (2005) Chemical Society of Japan.

**Figure 9:**
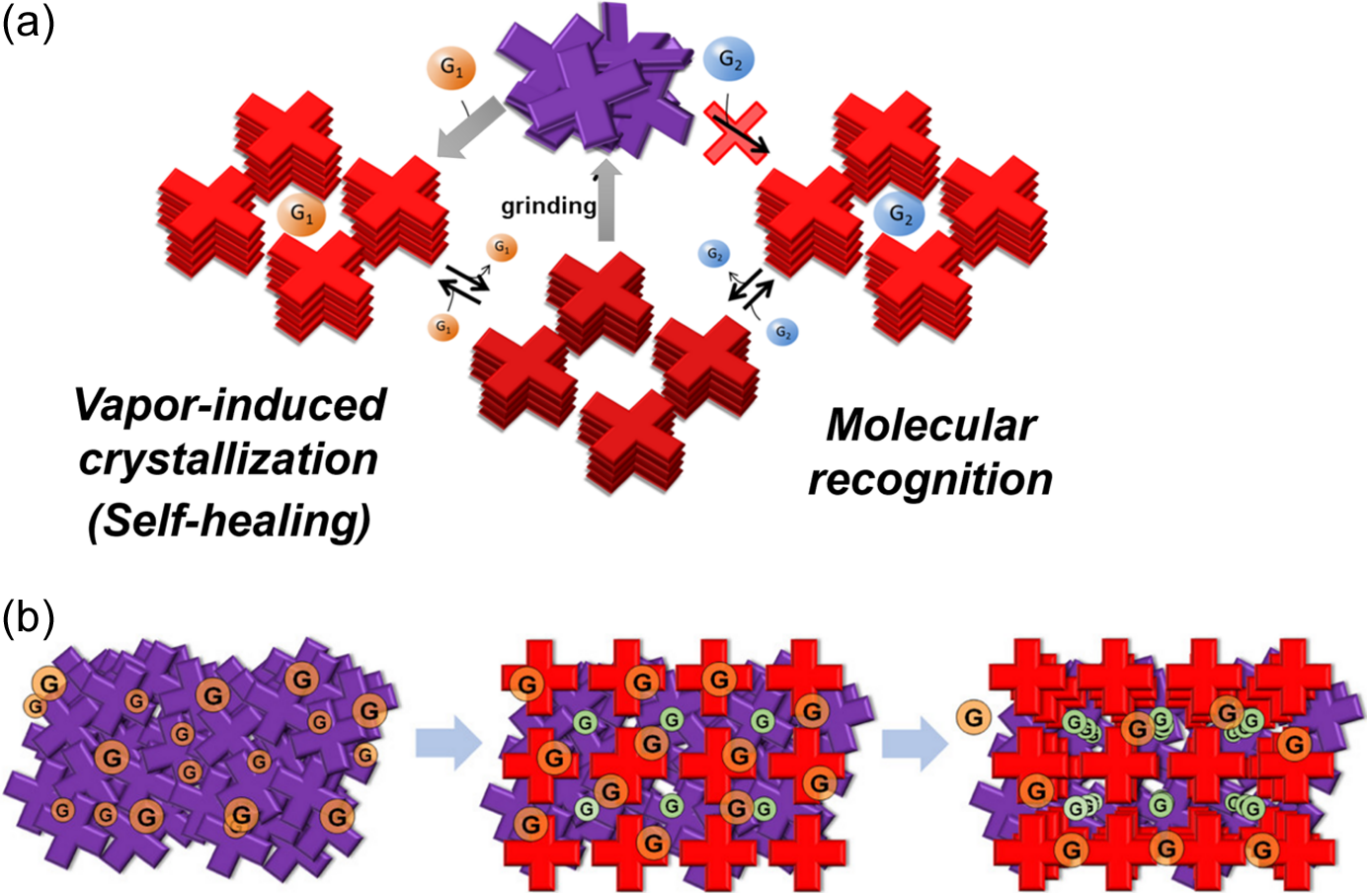
Schematic of the vapochromic behavior of [Pt(CN)_2_(H_2_dcbpy)]. (*a*) Cycle of vapor exposure, heating and grinding, including molecular recognition. (*b*) Stepwise process from the surface to the inside of the microparticles during vapor-induced crystallization. Reprinted (adapted) with permission from Shigeta *et al.* (2016 ▸). Copyright (2016) John Wiley & Sons. Part (*b*) reprinted (adapted) with permission from Ishii *et al.* (2021 ▸). Copyright (2021) American Chemical Society.

The introduction of a carb­oxy group, a typical supramolecular synthon, to [Pt(bpy)(CN)_2_] enabled the formation of a supramolecular structure with pores, which were constructed with Pt⋯Pt interactions and hydrogen bonds. Fig. 8 ▸ shows the packing structure of the [Pt(CN)_2_(H_2_dcbpy)] (H_2_dcbpy = 4,4′-di­carb­oxy-2,2′-bi­pyridine) complex (Kato *et al.*, 2005 ▸). The vapochromic behaviors of the analogous supramolecular crystal [Pt(CN)_2_(H_2_dcphen)] (H_2_dcphen = 4,7-di­carb­oxy-1,10-phenanthroline) were mechanically controlled with this complex because the supramolecular framework is sufficiently robust to maintain the structure through the desorption/absorption processes of vapor molecules; however, this framework is easily destroyed by manual grinding (Shigeta *et al.*, 2016 ▸). The scheme in Fig. 9 ▸(*a*) shows that the resulting purple amorphous form reverts to the red crystalline form when exposed to a vapor such as MeOH and is retained after desorption of the guest molecules. An empty framework can absorb other guest molecules that cannot be absorbed in the amorphous form. The vapor-induced crystallization of the Pt complex was recently analyzed using confocal laser phospho­rescence microscopy (Ishii *et al.*, 2021 ▸). Consequently, the vapor-induced crystallization mechanism was explained as a stepwise process from the surface to the interior [Fig. 9 ▸(*b*)]. When vapor molecules (methanol in this case) are adsorbed onto amorphous particle surfaces, the adsorbed MeOH molecules initiate crystallization on the surface, which generates voids. The voids generated on the surface enable the MeOH molecules to infiltrate the particles, thus leading to the crystallization inside. Therefore, the construction of a porous framework accelerates the vapor-induced crystallization.

### 2.4. Chromic triboluminescence

Triboluminescence (TL), or mechanoluminescence (ML), is a luminescence phenomenon induced via mechanical stimuli. TL is interesting because the luminescence is observed without photo-excitation, similar to ordinary photoluminescence (PL). The TL phenomenon has been known for a long time (*e.g.* light emission when quartz crystals are struck together like flints). TL has attracted considerable attention from many researchers owing to an increasing number of strongly luminescent materials (Jha & Chandra, 2014 ▸; Karimata & Khusnutdinova, 2022 ▸; Gu *et al.*, 2023 ▸; Shohag, 2023 ▸).

**Figure 10:**
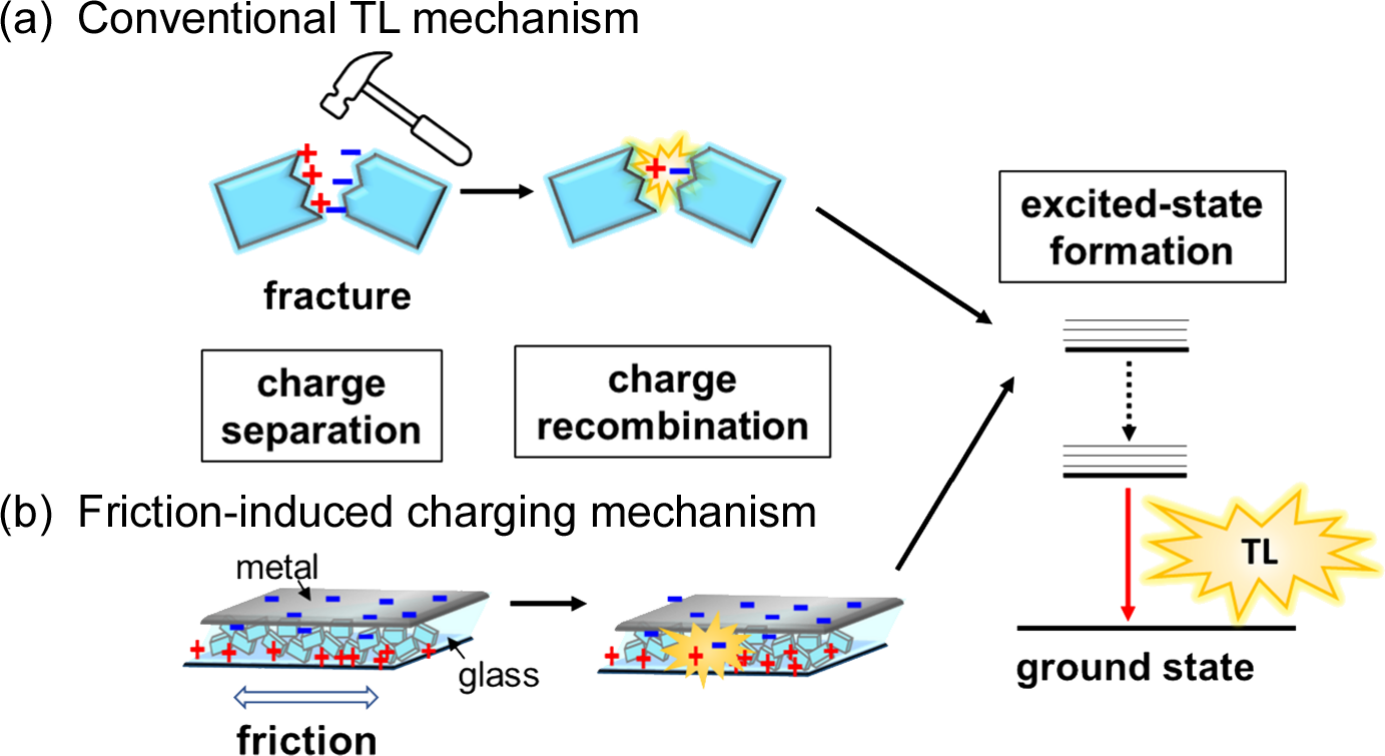
(*a*) Conventional TL mechanism based on crystal fractures and (*b*) friction-induced TL mechanism. Reproduced from Sasaki *et al.* (2023 ▸) with permission from the Royal Chemical Society.

Conventionally, the TL phenomenon was attributed to the piezoelectric effect of non-centrosymmetric crystals. The TL of crystals comprising intrinsic non-luminescent materials, such as inorganic salts and sucrose, was attributed to the emission from N_2_ gas in the air. For emissive materials, secondary emission was possible due to the excitation by the radiation from N_2_ gas, which was called tribophotoluminescence (TPL) (Sweeting, 2001 ▸). However, with an increasing number of highly photoluminescent materials, several cases of TL occurred via direct excitation. Fig. 10 ▸(*a*) shows a simplified scheme of TL, where efficient charge separation is induced under mechanical stimuli, followed by charge recombination, thus resulting in excited-state formation. Once an excited state is generated, luminescence can occur, similar to PL. Further, the TL materials must exhibit efficient radiative decay. Bright TL has been reported in luminescent metal complex crystals of europium(III), manganese(II) and copper(I) complexes. Many crystals exhibiting bright TL belong to the non-centrosymmetric space group, consistent with the piezoelectric mechanism. However, TL could be observed regardless of whether the crystal was centrosymmetric or non-centrosymmetric, and further mechanistic investigation was required (Wong *et al.*, 2017 ▸; Karimata & Khusnutdinova, 2022 ▸). Very recently, Hirai *et al.* (2023 ▸) reported extremely weak PL but strong TL for a dinuclear Eu(III)-β-diketonato complex bridged by a rigid and bent organic linker, [Eu_2_(tmh)_6_(dpdf)] [where tmh = 2,2,6,6-tetra­methyl­heptane-3,5-dionate(1−) and dpdf = 2,7-bis­(di­phenyl­phospho­ryl)-9,9-di­methyl­fluorene]. The tmh ligand quenched the PL through an efficient quenching process via the LMCT (tmh to Eu^3+^) state. However, the TL that did not undergo the quenching process became active.

**Figure 11:**
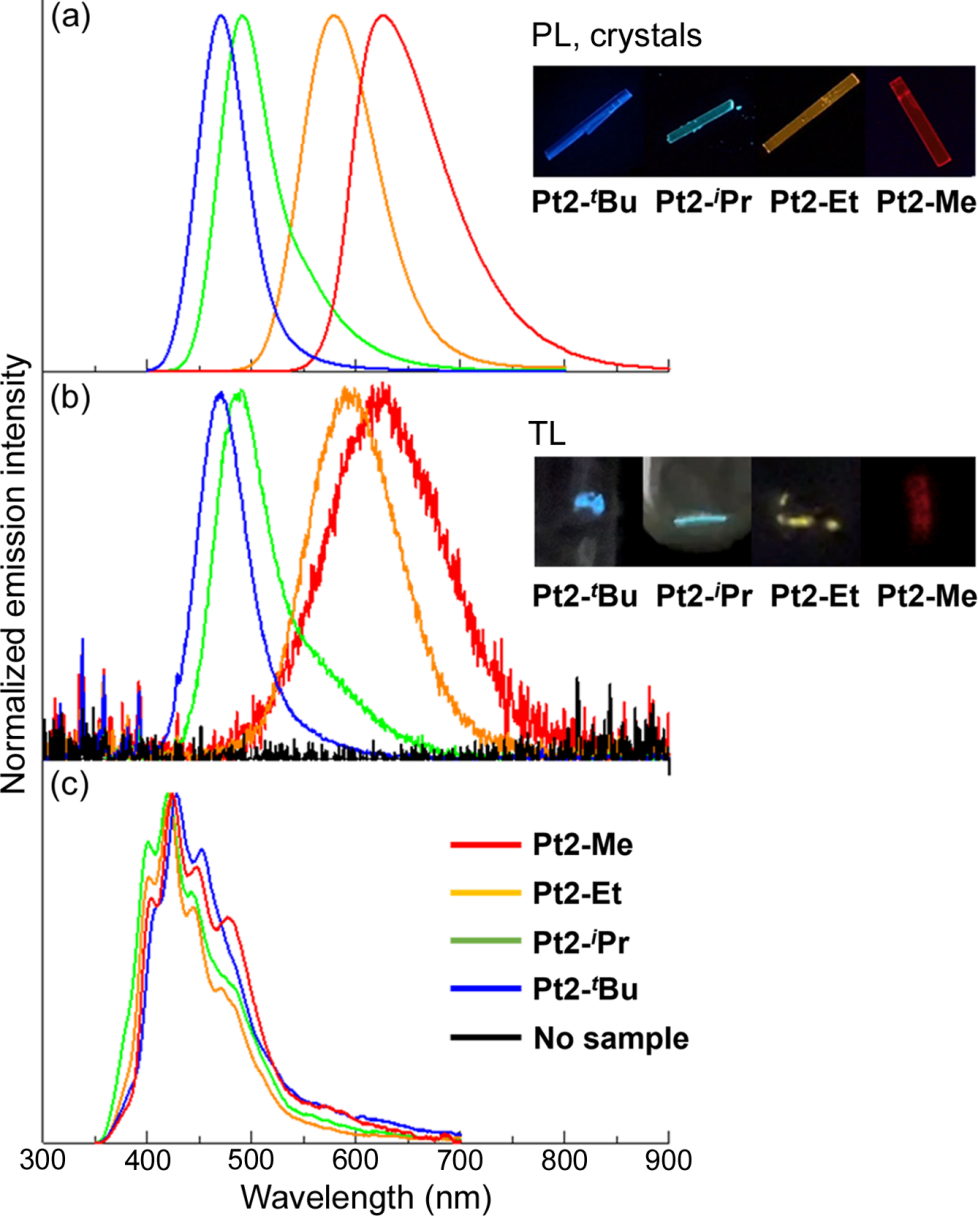
(*a*) PL (λ_ex_ = 350 nm) and (*b*) TL spectra of four [Pt(CN)_2_(Rim-Mepy)] complexes and their corresponding photographs. Red: Pt2-Me, orange: Pt2-Et, green: Pt2-*^i^*Pr and blue: Pt2-*^t^*Bu. The black line represents the background signal without the sample. (*c*) PL (λ_ex_ = 280 nm) of the complex (2.5 × 10^−7^ *M*) in MeOH–EtOH (1:1 *v*/*v*) at 77 K. Reproduced from Sasaki *et al.* (2023 ▸) with permission from the Royal Chemical Society.

**Figure 12:**
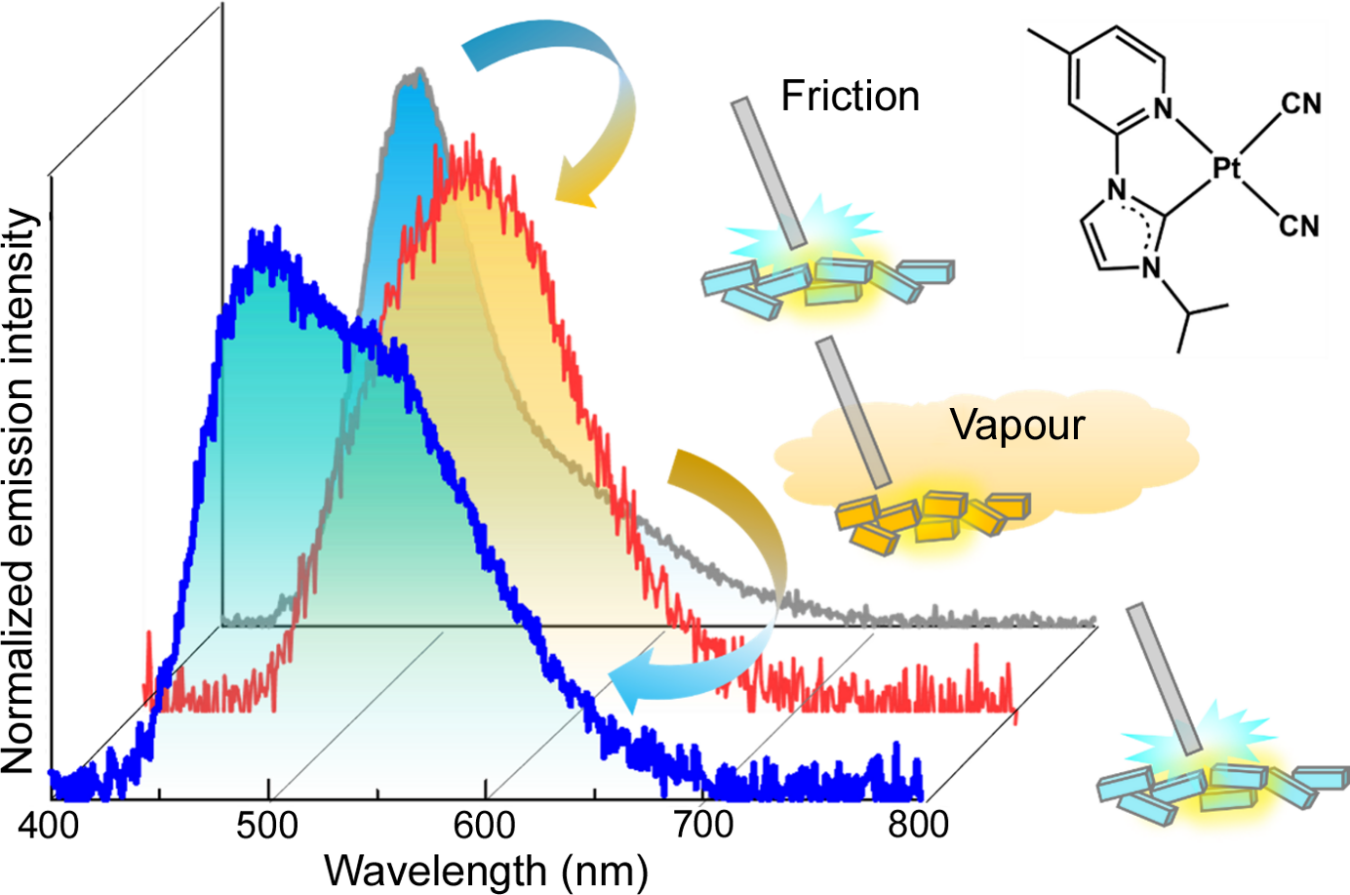
Chromic TL of Pt2-*^i^*Pr. Reproduced from Sasaki *et al.* (2023 ▸) with permission from the Royal Chemical Society.

Our group recently reported another series of Pt(II)-NHC complexes (as described in Section 2.1) with the general formula [Pt(CN)_2_(*R*im-Mepy)] [where *R*im-Mepy = 1-alkyl-3-(4-methyl-pyridin-2-yl)-1*H*-imidazolium; and *R* = Me (Pt2-Me), Et (Pt2-Et), *^i^*Pr (Pt2-*^i^*Pr) and *^t^*Bu (Pt2-*^t^*Bu)], which exhibited light emission by scratching the samples in the dark (Sasaki *et al.*, 2023 ▸). Although the TL of a relevant Pt(II)-NHC complex has been reported previously (Hsu *et al.*, 2016 ▸), the new series of Pt(II) complexes is unique owing to the observation of chromic behavior of TL and PL. The Pt(II) complexes exhibited TL with different colors when the crystalline powder samples in the Schlenk tube were scratched with a stainless-steel spatula. The TL spectra were consistent with the PL spectra, indicating that the emission origin is from the ^3^MMLCT state, as reported in the previous series (see Section 2.1; Fig. 11 ▸). The TL intensity increased with decreasing pressure for all the complexes, independent of the crystal space group. Me-, *^i^*Pr- and *^t^*Bu-substituted complexes adopted centrosymmetric space groups (*Pbcm*, *P*1, *P*1, respectively), whereas the Et-substituted form revealed the *P*2_1_2_1_2_1_ space group. These findings suggest a TL mechanism different from that based on the piezoelectric effect. Among the series, the Pt-*^i^*Pr complex exhibits distinct mechanochromic luminescence, and the color of the PL changes from blue to yellow when the crystal sample is ground (Fig. 12 ▸). Powder X-ray diffraction studies indicate amorphization of the sample by grinding. When the ground sample is exposed to DMF vapor, the original light-blue color is recovered, and the reversible behavior of the color changes in repeated mechano- and vapo-chromic cycles is observed, indicating that it is a unique chromic system for both PL and TL. Taken together with other experimental results, we conclude that the TL emission of the Pt(II) complexes is due to the friction-induced charging mechanism [Fig. 11 ▸(*b*)], wherein charge separation occurs between the glass Schlenk flask and the stainless-steel spatula, followed by charge recombination on the sample, thus generating an excited state. The increase in the TL intensity under reduced pressure supported this mechanism as it could be attributed to the suppression of gas discharge, resulting in the efficient formation of excited states of the Pt(II) complexes. A similar friction-induced charging mechanism for TL was recently reported by Karimata *et al.* (2022 ▸) using polymer films. They successfully prepared TL films by blending conventional luminescent molecules such as pyrene and [Ir(ppy)_3_] with poly(methyl methacrylate).

Thus, the expanded TL chemistry revealed that TL is a general phenomenon with great potential for various mechanical sensor applications. In particular, the chromic phenomenon of TL in the assembled Pt(II) complexes could be beneficial for evaluating the strength of the mechanical stimuli via color in situations where quantitative evaluation of TL efficiency by TL intensity is not easy.

## 3. Luminescent Pt(II) complexes controlled by counter ions

### 3.1. Highly efficient luminescence

Pt(II) complexes are extremely sensitive to the environment because of their square-planar structures. Intermolecular interactions can cause non-radiative deactivation, such as in aromatic organic fluoro­phores, termed aggregation-caused quenching. The primary strategy for overcoming this problem is sophisticated molecular design. Conformational and/or orientational control to cut non-radiative deactivation enabled the aggregation-induced luminescence for various organic crystals (Cai & Liu, 2020 ▸; Zhao *et al.*, 2020 ▸). In Pt(II) complexes, rigid structures with tetradentate ligands such as N^C-O-LL′ (Turner *et al.*, 2013 ▸) (see Scheme[Chem scheme1]) were found to be effective for highly efficient emission quantum yield, and various Pt(II) complexes with tetradentate ligands bearing bulky substituents were developed as efficient phospho­rescent emitters in organic light-emitting diodes (OLEDs) (Hang *et al.*, 2013 ▸; Kui *et al.*, 2013 ▸; Park *et al.*, 2022 ▸; Li *et al.*, 2022 ▸).

Another strategy involves the precise control of intermolecular interactions. As mentioned in Section 2, Pt(II)–Pt(II) interactions effectively construct highly ordered assemblies with characteristic emission states. The OLED device using the square-planar Pt(II) complex [Pt(fppz)_2_] [where fppz = 5-(pyridin-2-yl)-3-(tri­fluoro­methyl)-1*H*-pyrazol-1-ide] exhibited a remarkably high emission quantum yield of 96% and external quantum efficiency (EQE) of 38.8%, which exceeded the conventional theoretical value using emitters of isomorphous orientation (∼20%) (Kim *et al.*, 2016 ▸). The high EQE is attributed to the perfect regulation of the emitting dipole orientation by arranging the molecular orientation which enhanced the out-coupling efficiency of the light emitted in the device.

### 3.2. Luminescence of discrete Pt(II) complex anions in crystals

**Figure 13:**
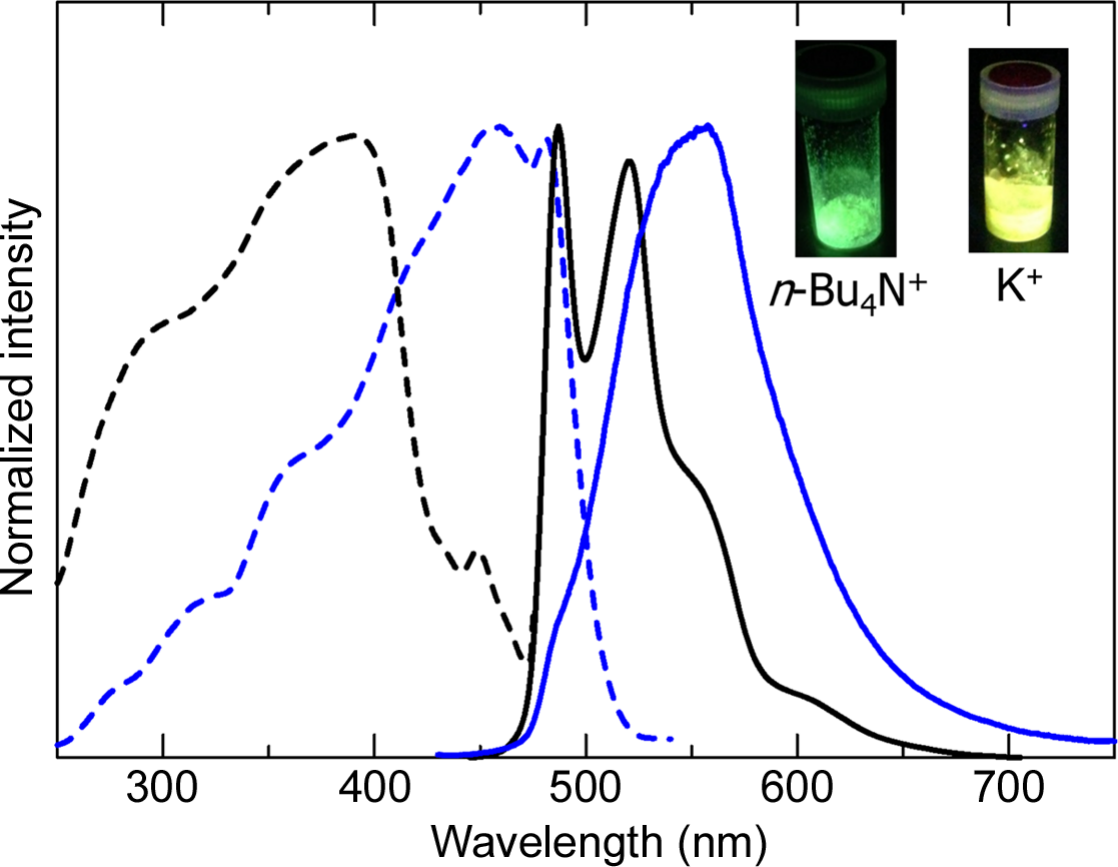
Emission and excitation spectra of the *n*-Bu_4_N^+^ (black lines) and K^+^ (blue lines) salts of the anionic complex [Pt(CN)_2_(*p*tpy)]^−^ at room temperature. Reproduced from Ogawa *et al.* (2015 ▸) with permission from the Royal Chemical Society.

**Figure 14:**
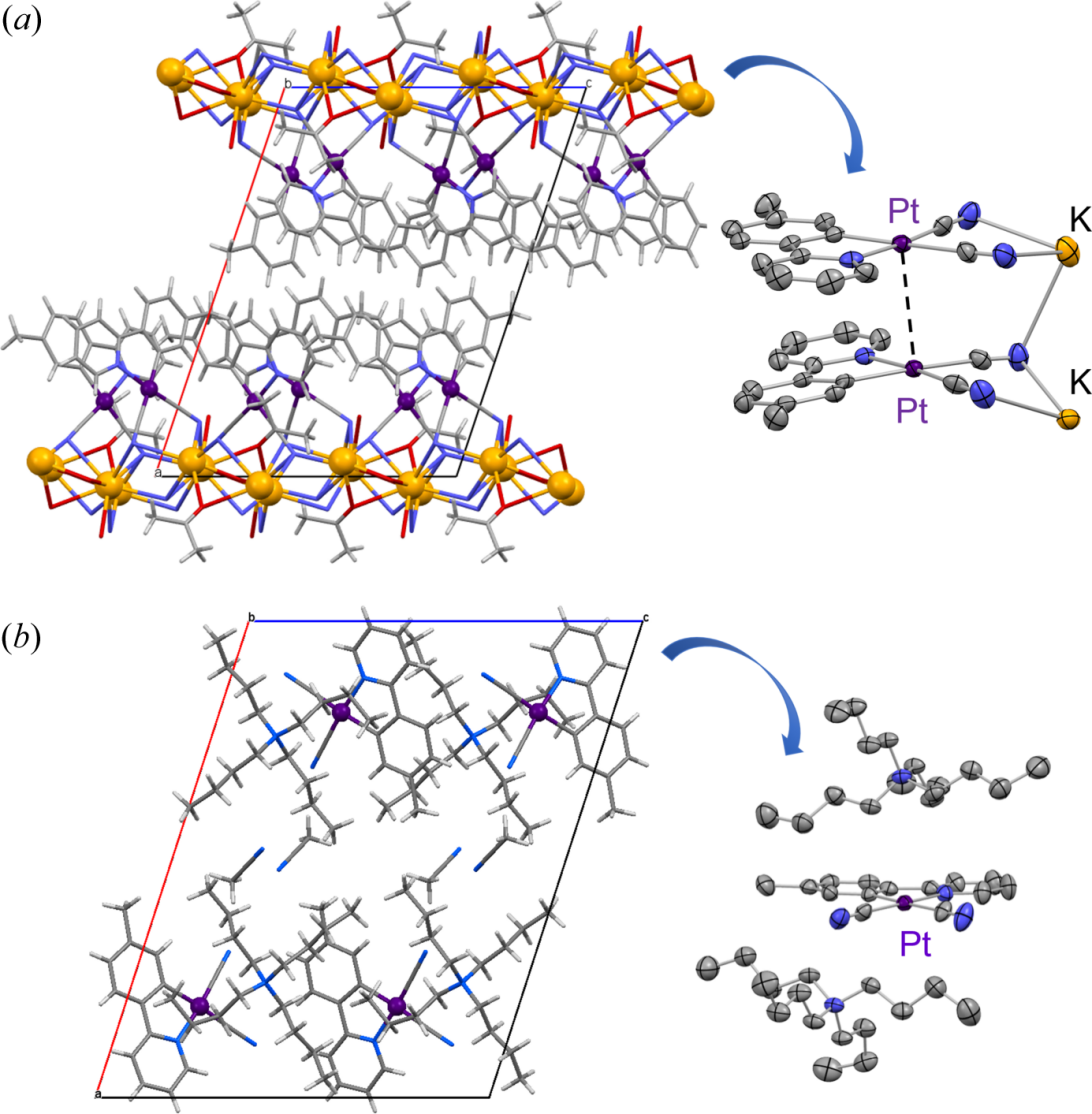
Packing structures of (*a*) K^+^ and (*b*) *n*-Bu_4_N^+^ salts of the anionic complex [Pt(CN)_2_(*p*tpy)]^−^. Reproduced from Ogawa *et al.* (2015 ▸) with permission from the Royal Chemical Society.

If metal complexes have a charge, counter ions act as another factor to control the intermolecular interactions and crystal space. When a cyclo­metalating ligand such as 2-phenyl­pyridinate (ppy) is used instead of neutral ligands such as bpy and *R*-impy, anionic Pt(II) complexes are formed. These crystal arrangements strongly depend on the counter cations, which affect the luminescence properties. For example, the Pt(II) complex [Pt(CN)_2_(*p*tpy)]^−^ [where *p*tpy = 5-methyl-2-(pyridin-2-yl)phenyl] exhibited different luminescence features depending on the counter cations (Ogawa *et al.*, 2015 ▸). The potassium salt of the complex exhibited yellow luminescence with moderate intensity (Φ = 0.09) at room temperature. However, on replacing K^+^ with the *n*-Bu_4_N^+^ ion, a highly luminescent solid exhibiting green emission with a very high emission quantum yield (Φ = 0.72) was obtained. Fig. 13 ▸ shows the emission and excitation spectra of the two salts of this complex at room temperature. The *n*-Bu_4_N^+^ salt exhibits the emission spectrum with a vibronic structure typical for the π–π* emission from the cyclo­metalating ligand (*p*-tolyl pyridine), indicating that the emission originated from the monomer complex. However, the potassium salt exhibits a broad spectrum, suggesting an influence of the intermolecular interactions. As shown in Fig. 14 ▸(*a*), a beautiful arrangement of the Pt(II) complex anions and the K cations can be seen in the crystal structure of the potassium salt. The Pt complex is dimerized with a short Pt⋯Pt distance [3.2785 (7) Å], suggesting that the yellow luminescence is the ^3^MMLCT emission based on the Pt–Pt electronic interaction. On the other hand, the *n*-Bu_4_N^+^ salt showed a completely different packing structure from that of the potassium salt. The Pt complex anions are located in a discrete space surrounded by *n*-Bu_4_N^+^ cations [Fig. 14 ▸(*b*)]. Therefore, intense luminescence originating from the ππ* state of the monomer complex could be realized by placing the Pt complex in a discrete and confined space. Based on this concept, several Pt(II) complex salts with bulky ammonium have been developed as highly luminescent materials (Berenguer *et al.*, 2007 ▸; Rausch *et al.*, 2010 ▸; Ogawa *et al.*, 2018*a* ▸;Yoshida & Kato, 2020 ▸).

### 3.3. Thermochromic luminescence of Pt(II) complex ionic liquid

**Figure 15:**
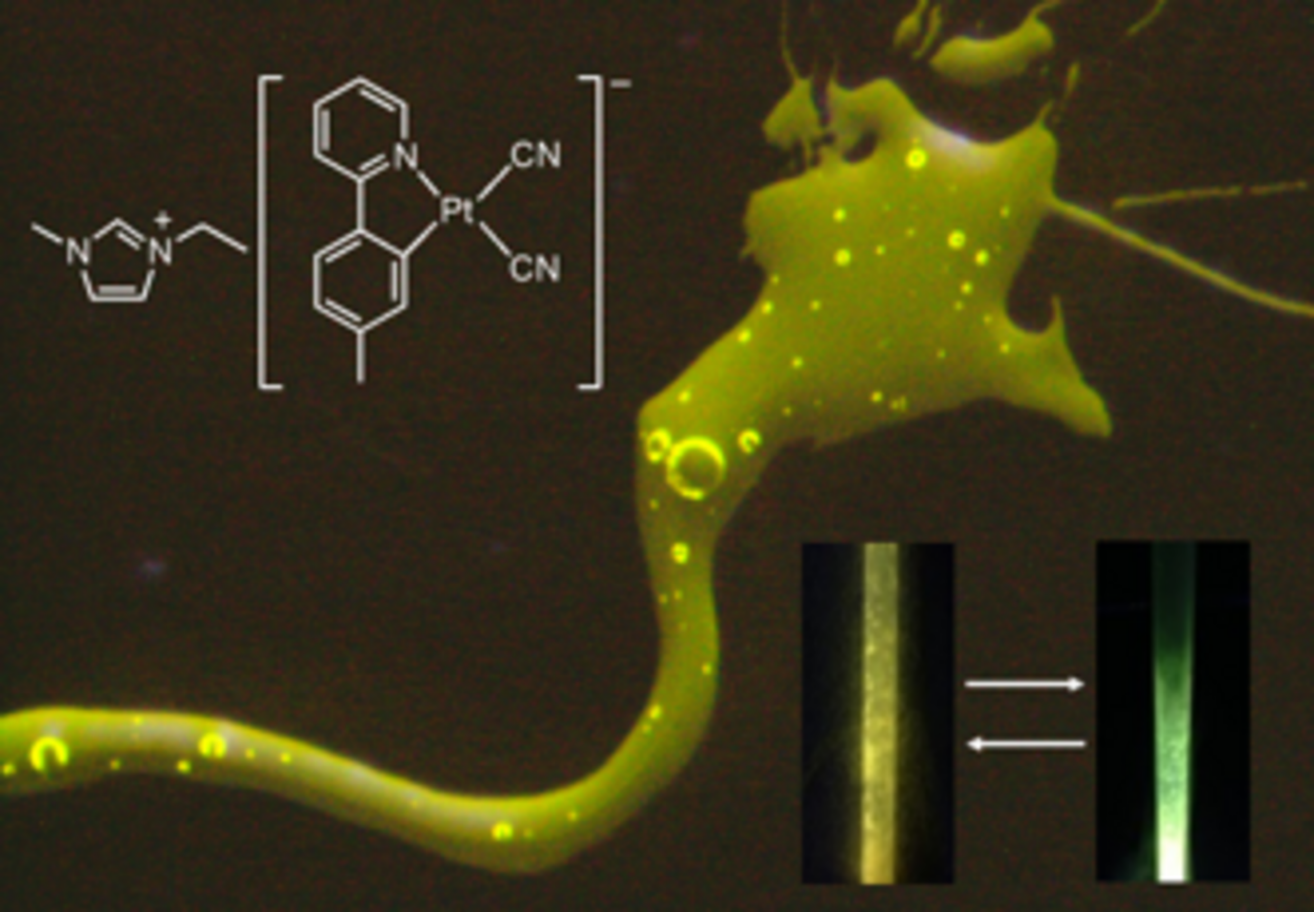
Luminescent thermochromic ionic liquid (C_2_mim)[Pt(CN)_2_(*p*tpy)]. Reproduced from Ogawa *et al.* (2015 ▸) with permission from the Royal Chemical Society.

Controlling the softness of the crystals is another effect of counter cations in anionic Pt(II) complexes, which enables a unique chromic behavior. By substituting the K ion of the previously mentioned K[Pt(CN)_2_(*p*tpy)] with the 1-ethyl-3-methyl­imidazolium ion (C_2_mim), a luminescent Pt(II) complex ionic liquid was obtained for the first time (Ogawa *et al.*, 2015 ▸; Ogawa *et al.*, 2018*b* ▸). This complex was a sticky liquid at room temperature that exhibited yellow luminescence on UV irradiation (Fig. 15 ▸). This ionic liquid exhibited interesting thermochromic luminescence behavior owing to the temperature-dependent energy transfer from the mononuclear to aggregated species. However, this ionic liquid was never crystallized and only showed a glass–liquid transition at −10°C.

### 3.4. Luminescence on–off switch based on supercooled liquid–crystal transformation

**Figure 16:**
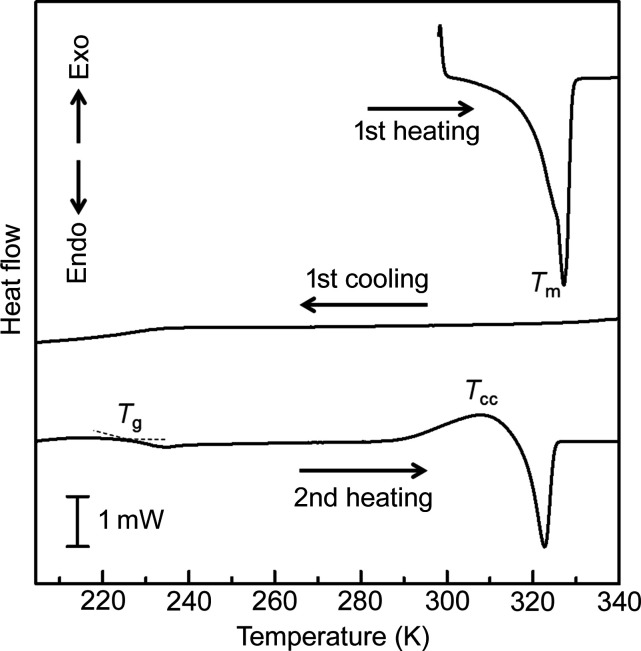
DSC curves of (P_6,6,6,14_)[Pt(ppy)Cl_2_] under N_2_ flow (scan rate = 5 K min^−1^). Reprinted with permission from Yoshida *et al.* (2022 ▸). Copyright (2022) John Wiley & Sons.

**Figure 17:**
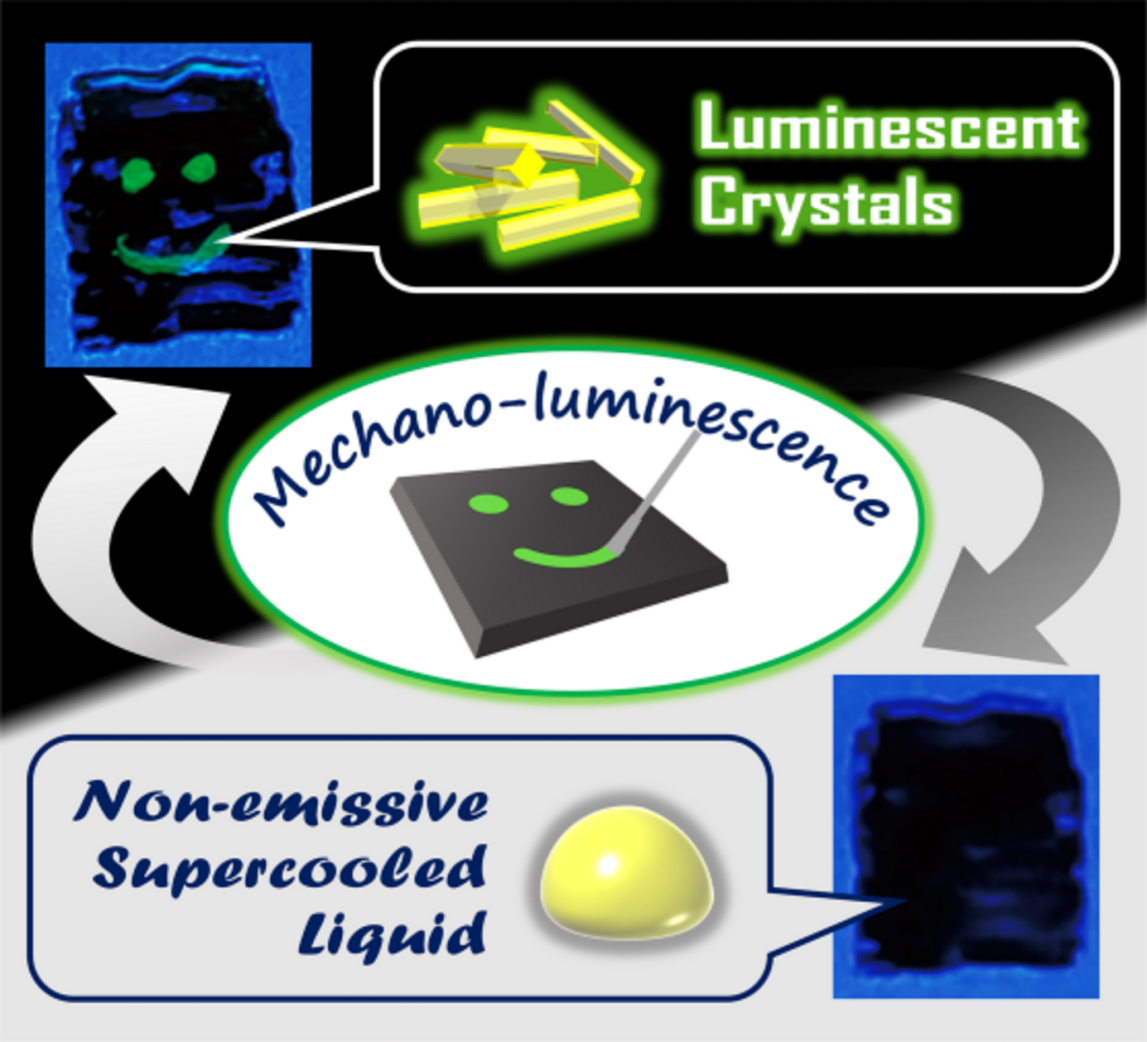
Photographs of the melted 2-Cl-film (P_6,6,6,14_)[Pt(ppy)Cl_2_] before and after scratching with a spatula. Reprinted with permission from Yoshida *et al.* (2022 ▸). Copyright (2022) John Wiley & Sons.

An alkyl phospho­nium cation with non-equivalent alkyl chains was used as a counter cation to fabricate an ionic liquid that could transform to a crystalline state. The complex, (P_6,6,6,14_)[Pt(ppy)Cl_2_] (where P_6,6,6,14_^+^ = tri­hexyl­tetra­decyl­phospho­nium) exhibited on–off switching via a supercooled liquid–crystal transition (Yoshida *et al.*, 2022 ▸). The differential scanning calorimetry (DSC) curve (Fig. 16 ▸) shows that the crystals of this complex melt at 326 K. Additionally, no crystallization is observed during the cooling process, and only a glass transition is observed at approximately 225 K, indicating that the supercooled liquid appears at room temperature. During the heating process of the glass sample, an exothermic peak is observed, indicating that crystallization occurs at approximately 300 K, and the crystals melt again at 323 K. The ionic liquid is non-emissive; however, it exhibits strong green luminescence after crystallization. Hence, the complex exhibits an interesting on–off switching luminescence behavior based on the phase transition between the supercooled liquid and the crystal. Moreover, this liquid–crystal transition was accelerated by mechanical stimuli and showed a rapid crystallization induced by mechanostress. If the thin film of this Pt(II) complex ionic liquid is scratched, the trace emits luminescence under UV light owing to crystallization, which reverts to the original non-luminescent film by heating to temperatures higher than the melting point (326 K), as shown in Fig. 17 ▸. Therefore, various luminescence properties of Pt(II) complexes were developed and investigated using different counter cations.

## 4. Conclusions

This article reviews the chromic luminescence of Pt(II) complexes in terms of photofunctional soft crystals, which are highly ordered crystalline materials exhibiting remarkable color changes in response to gentle stimuli such as vapor exposure and weak mechanical forces.

For Pt(II) complexes exhibiting assembly-induced luminescence, the control of luminescent colors was achieved by fine-tuning the stacking structures, resulting in intense luminescence over red–blue without changing the emission origin, the ^3^MMLCT emission. A reversible and stepwise SCSC transformation, accompanied by a remarkable luminescence color change, was observed in some crystals via water vapor adsorption and desorption. Remarkably, the dynamics of the crystal transformation that maintain single crystallinity were elucidated via *in situ* single-crystal diffraction measurements. Additionally, chromic TL was observed in similar types of assembled Pt(II) complexes during rubbing and vapor exposure, along with the corresponding intense PL.

Unique luminescence properties controlled by counter ions were induced for anionic Pt(II) complexes. Highly luminescent crystals were obtained by the formation of isolated spaces using bulky cations such as tetra(*n*-butyl) ammonium ions, whereas luminescent ionic liquids were constructed using low-symmetry organic cations such as ethyl methyl imidazolium ions. Mechano-triggered luminescence on–off switching was achieved using an inequivalent phospho­nium ion, which was based on regulating the supercooled liquid and bright luminescent crystalline phases.

Thus, the diverse and flexible crystal structures of Pt(II) complexes, as stable and designable structural units, are promising not only as simple luminescent materials but also as luminescent chromic materials. To date, luminescent Pt(II) complexes, together with other molecular luminescent dyes, have been used in OLEDs and environmental sensors that detect various gasses and volatile organic compounds. Assembly-induced luminescence of Pt(II) complexes based on Pt⋯Pt interactions have also been actively studied for environmental sensors since around 2000, although their applications are still in their infancy. Meanwhile, quantum dots, nano crystals of inorganic compounds, have seen remarkable progress in recent years. Nanocrystallization will also expand the application possibilities of Pt(II) complexes. In fact, it has been strongly suggested that the excited states of the assembled Pt(II) complexes are formed from trimeric and tetrameric Pt(II) complexes (Nakagaki *et al.*, 2020 ▸), which makes nanocrystalline materials promising for use in electronic devices, and fabric- or polymer-supported photofunctional materials. Furthermore, from the viewpoint of controlling metal–metal interactions, Pd(II) complexes with isomorphous 4*d* electronic systems can also be used. Mixed crystals of Pd and Pt are being investigated for their color control and highly efficient luminescence.

## References

[bb1] Aliprandi, A., Mauro, M. & De Cola, L. (2016). *Nat. Chem.* **8**, 10–15. 10.1038/nchem.2383 26673259

[bb2] Berenguer, J., Lalinde, E. & Torroba, J. (2007). *Inorg. Chem.* **46**, 9919–9930. 10.1021/ic701298z 17927171

[bb3] Cai, X. & Liu, B. (2020). *Angew. Chem. Int. Ed.* **59**, 9868–9886. 10.1002/anie.202000845 32128951

[bb4] Connick, W. B., Henling, L. M. & Marsh, R. E. (1996*a*). *Acta Cryst.* B**52**, 817–822.

[bb5] Connick, W. B., Henling, L. M., Marsh, R. E. & Gray, H. B. (1996*b*). *Inorg. Chem.* **35**, 6261–6265.

[bb6] Dutta, B., Ahmed, F. & Mir, M. H. (2022). *Cryst. Growth Des.* **22**, 6366–6378.

[bb7] Gu, J., Li, Z. & Li, Q. (2023). *Coord. Chem. Rev.* **475**, 214872.

[bb8] Hang, X. C., Fleetham, T., Turner, E., Brooks, J. & Li, J. (2013). *Angew. Chem. Int. Ed.* **52**, 6753–6756. 10.1002/anie.201302541 23658039

[bb9] Hirai, Y., Van Baaren, S., Ohmura, T., Nakanishi, T., Takeda, T., Kitagawa, Y., Hasegawa, Y., Métivier, R. & Allain, C. (2023). *Adv. Opt. Mater.* **11**, 2203139.

[bb10] Hsu, C. W., Ly, K. T., Lee, W. K., Wu, C. C., Wu, L. C., Lee, J. J., Lin, T. C., Liu, S. H., Chou, P. T., Lee, G. H. & Chi, Y. (2016). *Appl. Mater. Interfaces*, **8**, 33888–33898. 10.1021/acsami.6b12707 27960361

[bb11] Ishii, K., Takanohashi, S., Karasawa, M., Enomoto, K., Shigeta, Y. & Kato, M. (2021). *J. Phys. Chem. C*, **125**, 21055–21061.

[bb12] Jha, P. & Chandra, B. P. (2014). *Luminescence*, **29**, 977–993. 10.1002/bio.2647 24753157

[bb13] Karimata, A., Fayzullin, R. R. & Khusnutdinova, J. R. (2022). *ACS Macro Lett.* **11**, 1028–1033. 10.1021/acsmacrolett.2c00348 35905142

[bb14] Karimata, A. & Khusnutdinova, J. R. (2022). *Dalton Trans.* **51**, 3411–3420. 10.1039/d1dt04305f 35142308

[bb15] Kato, M. (2007). *Bull. Chem. Soc. Jpn*, **80**, 287–294.

[bb16] Kato, M. & Ishii, K. (2023). *Soft Crystals Flexible: Repose Systems with High Structural Order*. Springer. 10.1002/chem.201805641 PMC6593753 30653768

[bb17] Kato, M., Ito, H., Hasegawa, M. & Ishii, K. (2019). *Chem. A Eur. J.* **25**, 5105–5112. 10.1002/chem.201805641 PMC6593753 30653768

[bb18] Kato, M., Kishi, S., Wakamatsu, Y., Sugi, Y., Osamura, Y., Koshiyama, T. & Hasegawa, M. (2005). *Chem. Lett.* **34**, 1368–1369.

[bb19] Kato, M., Kosuge, C., Morii, K., Ahn, J. S., Kitagawa, H., Mitani, T., Matsushita, M., Kato, T., Yano, S. & Kimura, M. (1999). *Inorg. Chem.* **38**, 1638–1641.

[bb20] Kato, M., Yoshida, M., Sun, Y. & Kobayashi, A. (2022). *J. Photochem. Photobiol. Photochem. Rev.* **51**, 100477.

[bb21] Kim, K. H., Liao, J. L., Lee, S. W., Sim, B., Moon, C. K., Lee, G. H., Kim, H. J., Chi, Y. & Kim, J. J. (2016). *Adv. Mater.* **28**, 2526–2532. 10.1002/adma.201504451 26833629

[bb22] Kishi, S. & Kato, M. (2002). *Mol. Cryst. Liq. Cryst.* **379**, 303–308.

[bb23] Kui, S. C., Chow, P. K., Cheng, G., Kwok, C. C., Kwong, C. L., Low, K. H. & Che, C. M. (2013). *Chem. Commun.* **49**, 1497–1499. 10.1039/c2cc37862k 23321666

[bb24] Li, G., Liu, S., Sun, Y., Lou, W., Yang, Y.-F. & She, Y. (2022). *J. Mater. Chem. C.* **10**, 210–218.

[bb25] Makino, Y., Yoshida, M., Hayashi, S., Sasaki, T., Takamizawa, S., Kobayashi, A. & Kato, M. (2023). *Dalton Trans.* **52**, 8864–8872. 10.1039/d3dt00192j 36847788

[bb26] Martínez-Junquera, M., Lalinde, E. & Moreno, M. T. (2022). *Inorg. Chem.* **61**, 10898–10914. 10.1021/acs.inorgchem.2c01400 PMC9348835 35775932

[bb27] Morimoto, T., Yoshida, M., Sato–Tomita, A., Nozawa, S., Takayama, J., Hiura, S., Murayama, A., Kobayashi, A. & Kato, M. (2023). *Chem. A Eur. J.* **29**, e202301993. 10.1002/chem.202301993 37581259

[bb28] Nakagaki, M., Aono, S., Kato, M. & Sakaki, S. (2020). *J. Phys. Chem. C*, **124**, 10453–10461.

[bb29] Ni, J., Guo, Z., Zhu, Q., Liu, S. & Zhang, J. (2023). *Dyes Pigments*, **217**, 111406.

[bb30] Novoa, J. J., Aullon, G., Alemany, P. & Alvarez, S. (1995). *J. Am. Chem. Soc.* **117**, 7169–7171.

[bb31] Ogawa, T., Sameera, W. M. C., Saito, D., Yoshida, M., Kobayashi, A. & Kato, M. (2018*a*). *Inorg. Chem.* **57**, 14086–14096. 10.1021/acs.inorgchem.8b01654 30354093

[bb32] Ogawa, T., Sameera, W. M. C., Yoshida, M., Kobayashi, A. & Kato, M. (2018*b*). *Dalton Trans.* **47**, 5589–5594. 10.1039/c8dt00651b 29589867

[bb33] Ogawa, T., Yoshida, M., Ohara, H., Kobayashi, A. & Kato, M. (2015). *Chem. Commun.* **51**, 13377–13380. 10.1039/c5cc04407c 26207770

[bb34] Park, H. J., Jang, J. H., Lee, J. H. & Hwang, D. H. (2022). *Appl. Mater. Interfaces*, **14**, 34901–34908. 10.1021/acsami.2c06891 35867806

[bb35] Pérez Paz, A., Espinosa Leal, L. A., Azani, M. R., Guijarro, A., Sanz Miguel, P. J., Givaja, G., Castillo, O., Mas–Ballesté, R., Zamora, F. & Rubio, A. (2012). *Chem. A Eur. J.* **18**, 13787–13799. 10.1002/chem.201201962 22987280

[bb36] Rausch, A. F., Monkowius, U. V., Zabel, M. & Yersin, H. (2010). *Inorg. Chem.* **49**, 7818–7825. 10.1021/ic100851b 20672835

[bb37] Saito, D., Galica, T., Nishibori, E., Yoshida, M., Kobayashi, A. & Kato, M. (2022). *Chem. A Eur. J.* **28**, e202200703. 10.1002/chem.202200703 35446453

[bb38] Saito, D., Ogawa, T., Yoshida, M., Takayama, J., Hiura, S., Murayama, A., Kobayashi, A. & Kato, M. (2020). *Angew. Chem. Int. Ed.* **59**, 18723–18730. 10.1002/anie.202008383 32666592

[bb39] Sasaki, K., Saito, D., Yoshida, M., Tanaka, F., Kobayashi, A., Sada, K. & Kato, M. (2023). *Chem. Commun.* **59**, 6745–6748. 10.1039/d3cc01525d 37194401

[bb40] Shigeta, Y., Kobayashi, A., Ohba, T., Yoshida, M., Matsumoto, T., Chang, H.-C. & Kato, M. (2016). *Chem. Eur. J.* **22**, 2682–2690. 10.1002/chem.201503247 26636566

[bb41] Shigeta, Y., Nomoto, T., Kato, M. & Mizuno, M. (2023). *Inorg. Chem.* **62**, 66–74. 10.1021/acs.inorgchem.2c02865 36543520

[bb42] Shohag, M. A. (2023). *Cryst. Res. Technol.* **58**, 2300206.

[bb43] Stoyanov, S. R., Villegas, J. M. & Rillema, D. P. (2003). *Inorg. Chem.* **42**, 7852–7860. 10.1021/ic030084n 14632501

[bb44] Sweeting, L. M. (2001). *Chem. Mater.* **13**, 854–870.

[bb45] Turner, E., Bakken, N. & Li, J. (2013). *Inorg. Chem.* **52**, 7344–7351. 10.1021/ic302490c 23750738

[bb46] Wenger, O. S. (2013). *Chem. Rev.* **113**, 3686–3733. 10.1021/cr300396p 23317416

[bb47] Wong, H. Y., Lo, W. S., Chan, W. T. K. & Law, G. L. (2017). *Inorg. Chem.* **56**, 5135–5140. 10.1021/acs.inorgchem.7b00273 28388037

[bb48] Xu, F. F., Zeng, W., Sun, M. J., Gong, Z. L., Li, Z. Q., Zhao, Y. S., Yao, J. & Zhong, Y. W. (2022). *Angew. Chem. Int. Ed.* **61**, e202116603. 10.1002/anie.202116603 35020259

[bb49] Yam, V. W., Au, V. K. & Leung, S. Y. (2015). *Chem. Rev.* **115**, 7589–7728. 10.1021/acs.chemrev.5b00074 26158432

[bb50] Yam, V. W.-W. & Cheng, Y.-H. (2022). *Bull. Chem. Soc. Jpn*, **95**, 846–854.

[bb101] Yoshida, M. & Kato, M. (2018). *Coord. Chem. Rev.* **355**, 101–115.

[bb51] Yoshida, M. & Kato, M. (2020). *Coord. Chem. Rev.* **408**, 213194.

[bb52] Yoshida, M., Makino, Y., Sasaki, T., Sakamoto, S., Takamizawa, S., Kobayashi, A. & Kato, M. (2021). *CrystaEngComm*, **23**, 5891–5898.

[bb53] Yoshida, M., Sääsk, V., Saito, D., Yoshimura, N., Takayama, J., Hiura, S., Murayama, A., Põhako–Esko, K., Kobayashi, A. & Kato, M. (2022). *Adv. Opt. Mater.* **10**, 2102614.

[bb54] Zhao, Z., Zhang, H., Lam, J. W. Y. & Tang, B. Z. (2020). *Angew. Chem. Int. Ed.* **59**, 9888–9907. 10.1002/anie.201916729 32048428

